# Recognition Mechanisms between a Nanobody and Disordered Epitopes of the Human Prion Protein: An Integrative Molecular Dynamics Study

**DOI:** 10.1021/acs.jcim.2c01062

**Published:** 2022-12-29

**Authors:** Luca Mollica, Gabriele Giachin

**Affiliations:** † Department of Medical Biotechnology and Translational Medicine, University of Milan, Segrate, 20090 Milan, Italy; ‡ Department of Chemical Sciences (DiSC), University of Padua, 35131 Padova, Italy

## Abstract

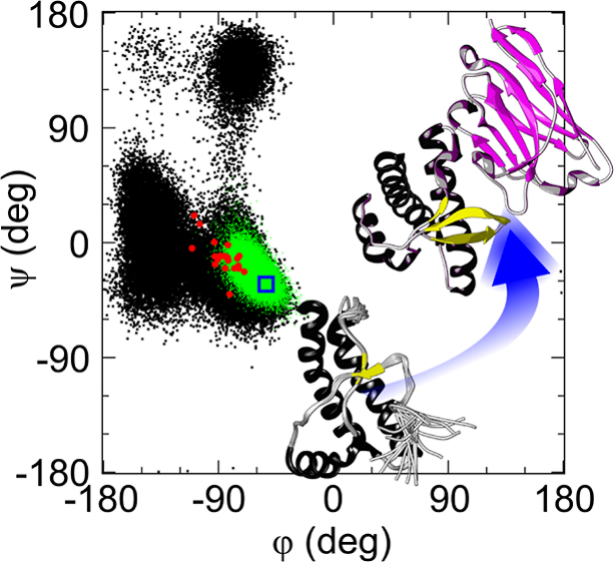

Immunotherapy using antibodies to target the aggregation of flexible proteins holds promise for therapeutic interventions in neurodegenerative diseases caused by protein misfolding. Prions or PrP^Sc^, the causal agents of transmissible spongiform encephalopathies (TSE), represent a model target for immunotherapies as TSE are prototypical protein misfolding diseases. The X-ray crystal structure of the wild-type (WT) human prion protein (HuPrP) bound to a camelid antibody fragment, denoted as Nanobody 484 (Nb484), has been previously solved. Nb484 was found to inhibit prion aggregation in vitro through a unique mechanism of structural stabilization of two disordered epitopes, that is, the palindromic motif (residues 113–120) and the β2−α2 loop region (residues 164–185). The study of the structural basis for antibody recognition of flexible proteins requires appropriate sampling techniques for the identification of conformational states occurring in disordered epitopes. To elucidate the Nb484-HuPrP recognition mechanisms, here we applied molecular dynamics (MD) simulations complemented with available NMR and X-ray crystallography data collected on the WT HuPrP to describe the conformational spaces occurring on HuPrP prior to Nb484 binding. We observe the experimentally determined binding competent conformations within the ensembles of pre-existing conformational states in solution before binding. We also described the Nb484 recognition mechanisms in two HuPrP carrying a polymorphism (E219K) and a TSE-causing mutation (V210I). Our hybrid approaches allow the identification of dynamic conformational landscapes existing on HuPrP and highly characterized by molecular disorder to identify physiologically relevant and druggable transitions.

## Introduction

Molecular recognition in protein–protein interactions is a fundamental phenomenon in biology underlying signalling, functional control and immune recognition. Understanding the key principles of protein binding to its partner has received considerable attention for drug discovery research targeting specific molecular interactions.^1^ The structural mechanisms that control ligand-induced protein conformational changes are described following the paradigms of conformational selection or induced fit models. In the conformational selection model, differing conformations involved in partner recognition already exist spontaneously in the absence of ligands, and conformational changes happen before ligand binding to produce a binding-competent state. Alternatively, in the induced fit model, ligands induce the partner to adopt their binding-competent conformation.^2−4^ The conformational selection paradigm plays a central role in pharmacological approaches targeting highly dynamic protein conformations, where potential interacting partners are able to stabilize a particular conformation from a spectrum of states possessing a desired biological activity.^5^

Proteins displaying a continuum of conformations lacking a well-defined structure under physiological conditions are denoted as intrinsically disordered (IDP) and are involved in cellular signalling and regulation, but also in pathological amyloidal aggregation processes associated with several human disorders, the most notable of which are neurodegenerative diseases.^6,7^ Parkinson’s disease, Alzheimer’s disease, and transmissible spongiform encephalopathies (TSE) are neurodegenerative diseases characterized by the amyloid accumulation of IDP proteins such as α-synuclein, amyloid-β (Aβ) peptide and prion protein, respectively.^8^ Antibodies can select and stabilize biologically significant conformations of monomeric amyloid proteins and prevent further aggregation, thus representing a promising tool for disease-modifying treatments thanks to their high specificity and binding affinity.^9−11^

**Figure 1:**
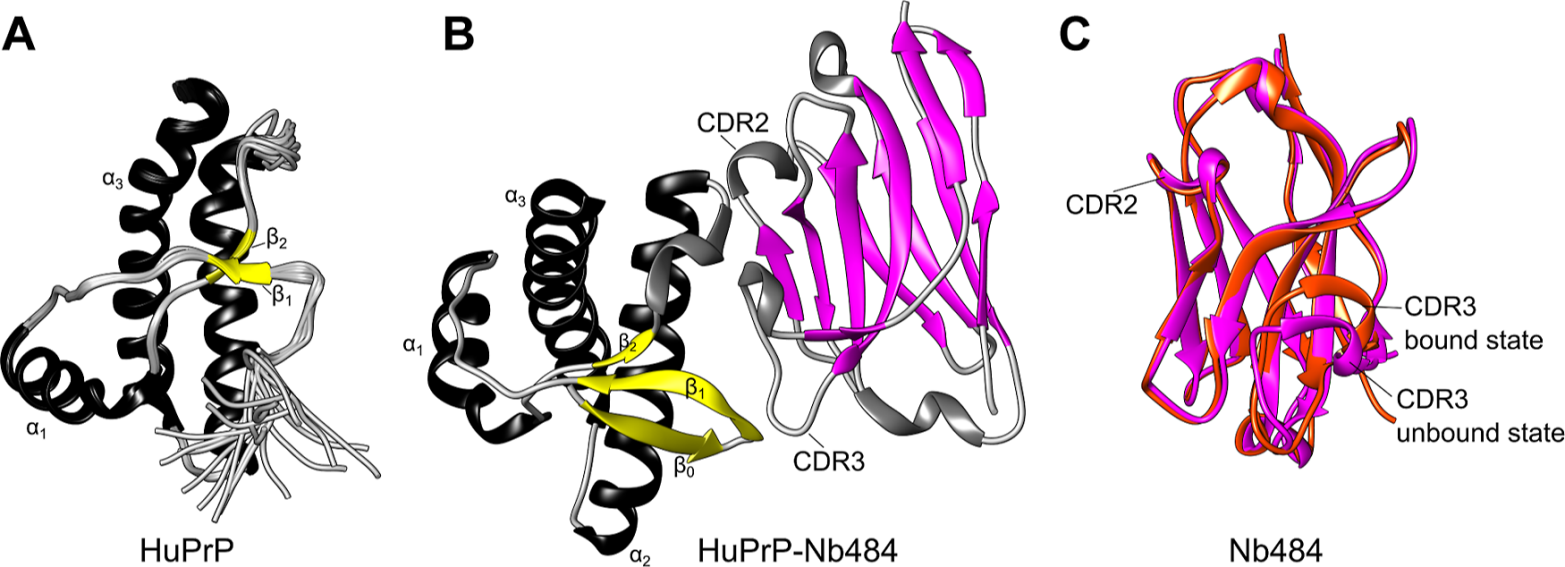
Cartoon representation of the NMR structure of WT HuPrP (residues 117–226, superposition of the 20 lowest-energy NMR structures, PDB ID 2lsb) in panel A and of the X-ray crystal structure of WT HuPrP (residues 117–226) bound to Nb484 (PDB ID 4kml) in panel B. The secondary structure motifs are highlighted and include the β1, α1, β2, α2, and α3 in free HuPrP and β0, β1, α1, β2, α2, and α3 in HuPrP bound to Nb484. The Nanobody binds to HuPrP through two complementarity-determining regions (CDR), named CDR2 and CDR3. Interacting surfaces on HuPrP side include the palindromic motif (residues 113–120) and the β2−α2 loop region (residues 164–185). In C, superposition of Nb484 X-ray crystal structures in the bound (PDB ID 4kml, coloured in red) and unbound states (PDB ID 6heq, coloured in magenta).

TSE, the prototypical protein misfolding disease, has long been recognised to have a protein-only self-propagating infectious mechanism that has represented a prominent target for immunotherapies during the last decades.^12^ Prions, or PrP^Sc^, are the proteinaceous infectious agent causing TSE in different mammalian species and derive from the post-translational conversion of the ubiquitously expressed cellular form of the prion protein, PrP^C^, into a misfolded, oligomeric, and neurotoxic form.^13,14^ The PrP^Sc^ neurotoxicity requires the interaction with its cellular counterpart PrP^C^, which ultimately leads to spongiform encephalopathy through a mechanism involving a hierarchy of distinct cellular events.^15^ The host-encoded PrP^C^ is a cell surface glycosylphosphatidylinositol (GPI)-anchored glycoprotein containing a flexible *N*-terminal moiety and a well-folded α-helical *C*-terminus.^16^ Detailed three-dimensional (3D) structures of human and other mammalian PrP^C^ have been solved by NMR (Figure 1A) and X-ray diffraction crystallography approaches.^17−19^ The first 3.14 Å resolution cryo-EM structure of infectious, brain hamster-derived PrP^Sc^ fibrils with *N*-linked glycans and a GPI anchor has been solved, unveiling a continuum of β-strand serpentine threading of the segment from residues 95–227 ^20^. Many other recent cryo-EM studies on PrP^Sc^ fibrils showed that these amyloids feature parallel in-register β-strand stacks.^21−25^

Thus far, the conformational conversion of a single PrP^C^ into infectious prions appears to be multi-step process whose molecular mechanisms remain still unclear. Interfering with key steps involved in prion conversion could facilitate the rational design of therapeutic strategies to prevent PrP^Sc^ formation.^26,27^ Application of anti-PrP^C^ antibodies suppresses prion replication in experimental animal models, presumably by stabilizing PrP^C^ conformation.^28^ Major drawbacks of immunotherapy to cure TSE are immune tolerance, neurotoxic side effects and their limited ability to cross the blood–brain barrier (BBB).^29−32^

Previously, the potential role of camelid heavy chain antibodies or Nanobodies (Nb) as anti-prion agents has been investigated.^33,34^ Differently from conventional antibodies, Nbs display superior properties such as small size, high stability, strong antigen-binding affinity, and the ability to cross the BBB.^35^ Nb-aided crystallography and Nb-aided cryo-EM represent key approaches in structural biology to study difficult protein targets and protein conformational states.^36,37^ It was found that a Nanobody, namely Nb484, is able to inhibit prion conversion in both cultured mouse neuronal cells and in Protein Misfolding Cyclic Amplification (PMCA) reactions^38^ without eliciting neurotoxic effects on organotypic cultured slices. Nb484 allowed the crystallization of both the full-length and *C*-terminal truncated forms of recombinant human prion protein (HuPrP) and mouse PrP. The high resolution X-ray crystal structures of HuPrP bound to Nb484 (HuPrP-Nb484) reveal a unique expansion of a β-sheet in the usually disordered *N*-terminal region containing the palindromic motif that, from residues ∼117 to 128, adopts a stable and structured antiparallel β-sheet configuration^33,34^ (Figure 1B). This palindromic “AGAAAAGA” motif (residues 113–120) plays an important role in the conversion to PrP^Sc^ and is likely to be involved in the PrP^C^–PrP^Sc^ interaction to initiate the conformational conversion.^39,40^ Additionally, Nb484 was found to bind to a second discontinuous and flexible epitope located inside the *C*-terminal folded domain, that is, the β2−α2 loop (residues 164–185), for which a correlation between its local structural variations and susceptibility to TSE has been described.^41−43^ While this loop shows local dynamic conformational changes in the unbound state, it adopts a rigid conformation when bound to Nb484.

Large conformational changes affect the PrP^C^ structure during its pathological conversion to PrP^Sc^. The study of HuPrP conformations trapped by the Nb484 may provide an important link to the conformational transitions in amyloid formation. Here, we provide a direct demonstration of conformational selection recognition mechanisms in WT HuPrP regions involved in the binding with Nb484. Using molecular dynamics (MD) simulations complemented with available X-ray crystallography and NMR data, we demonstrate that the palindromic motif and the β2−α2 loop of the free WT HuPrP populate similar folding conformations as those observed in the Nb-bound structure.

Additionally, we expanded our analysis to predict the Nb484-mediated recognition mechanisms in other two HuPrPs carrying disease-linked mutations, such as E219K and V210I, whose 3D structures in the unbound states were solved by solution-state NMR approaches.^44−46^ E219K is a naturally occurring HuPrP polymorphism found to modulate the susceptibility to Creutzfeldt-Jakob disease (CJD, the most common human prion disease form) depending on homozygosity or heterozygosity at codon 219: the allelic variants 219 K/K and 219 E/K are susceptible or resistant to PrP^Sc^ conversion, respectively.^47^ The hydrophobic mutation V210I is responsible to familial CJD, and it is one of the most common mutations observed among genetic TSE cases in the European population.^48,49^ The E219K and V210I NMR structures feature global 3D architectures similar to WT HuPrP;^16,44^ however, some specific local structural variations can be observed in these mutants with most pronounced differences involving the increased disorders of the β2−α2 loop.

Structural evidence from HuPrP pathological mutants, in which this loop region and the palindromic motif are involved in the spontaneous PrP^Sc^ conversion, supports the rationale for targeting these epitopes with a unique Nanobody able to trap these highly flexible segments.

We found that Nb484 mostly recognizes the flexible regions on WT and mutants HuPrP via a conformational selection mechanism. We also describe that the V210I mutant explores some additional conformations involving residues inside the β2−α2 loop that rarely converge into the Nb-bound state observed in the WT and E219K HuPrP. We interpret this difference proposing that for this mutant both conformational selection and induced fit recognition mechanisms play a role in the binding with Nb484. This study underlines the efficacy of Nb484 to stabilize the folding of aggregation-prone epitopes located on the HuPrP structure.

## Results and Discussion

### Conformational Dynamics of WT, E219K, and V210I HuPrP in the *apo* and *Holo* States

To investigate if the WT HuPrP in the Nb484-unbound state (*apo*) explores similar folding conformational trajectories as those observed in the Nb484-bound state (*holo*), we performed and analyzed an overall 3 μs all-atom MD simulation starting from its deposited NMR structure.^44^ We also included in the MD analysis the E219K polymorphism and the V210I pathological mutant.^44,46^ Similarly, a series of MD simulations (3 replica of 1 μs each) was carried out on the WT HuPrP bound to Nb484, previously obtained by X-ray diffraction (XRD) protein crystallography.^33,34^

**Figure 2:**
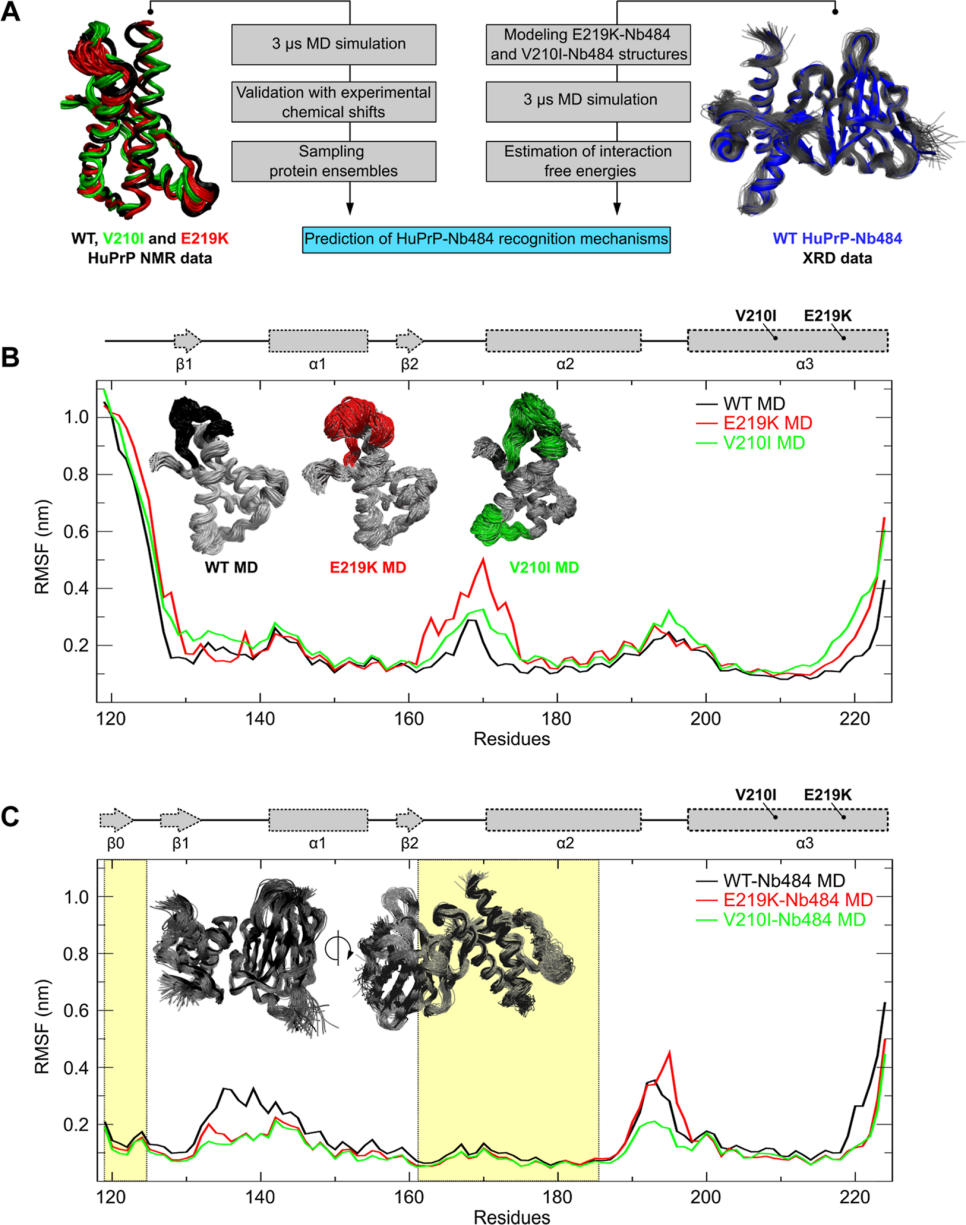
Overall computational flowchart and root mean square fluctuations (RMSFs) of *apo* and *holo* HuPrP structures. (A) Workflow to study the conformational energy landscapes of HuPrP by means of MD starting from available experimental NMR and X-ray diffraction (XRD) protein crystallography data. As NMR structures, we used the following PDB IDs: 2LSB (WT), 2LFT (E219K), and 2LEJ (V210I); as XRD structure, we used the PDB ID 4N9O (WT HuPrP-Nb484). (B) Analysis of RMSF in the MD structures. The regions that show higher flexibility are highlighted with a different colour in the MD 3D structures of WT and mutant HuPrP (see in the inset). The first and the last residues have a RMSF value higher than 0.3 nm since they are in a terminal position and are omitted here from the analysis. In black, green and red the RMSF of the WT, V210I, and E219K HuPrP MD structures, respectively. (C) Analysis of the 3 μs-RMSF trajectories of the WT, E219K, and V210I HuPrP-Nb484 complexes. The HuPrP regions involved in the Nb484 binding are highlighted in yellow color (residues 123 to 125 and from 164 to 185). In the inset, a representative MD ensemble of the WT HuPrP-Nb484. On the top, secondary structures of HuPrP indicate the positions of E219K and V210I mutations.

The E219K–Nb484 and V210I–Nb484 structures have not been solved yet. Although the NMR structures of these two HuPrP variants show increased flexibility in the β2−α2 loop, it is likely that the Nb484 may also bind these structures since the mutations are not located inside the two epitopes. Additionally, Nb484 was found to bind HuPrP through a distinct conformational change occurring at the complementarity-determining region, CDR2, which is a loop that recognizes the intrinsic flexibility of HuPrP epitopes (Figure 1C). In support, it has also been observed that disordered epitopes are as likely to be recognized by antibodies as ordered motifs, which are involved in antibody recognition by concave paratopes, a strategy that maximizes the extent and complementarity of the interaction.^50^ In order to include in this type of study also the HuPrP mutants and to face the unavailability of experimental structures of their complexes with Nb484, we modelled the structures of E219K and V210I *holo* forms on the basis of the available XRD structure of WT HuPrP–Nb484 and simulated using the same approach as for the WT *holo* form. The workflow describing the MD simulations carried out on the three HuPrP systems in both *apo* and *holo* forms is described in Figure 2A.

As previously reported,^51^ MD simulations’ reliability can be verified by means of an analytical and quantitative comparison of experimental data and simulated observables obtained by statistically meaningful ensembles of structures. Having these three HuPrP systems being studied in their *apo* forms by NMR, their computed dynamics can be directly compared to an experimental structure through the calculation of chemical shifts from the structures obtained as MD simulation frames. This allowed us to use an iterative procedure applying the code SPARTA+ to a selection of 3,000 frames of the overall 3 μs MD trajectories (i.e., with a sampling rate of 1 frame every 1 ns) in order to predict the chemical shifts of Cα and Cβ nuclei. The root mean square deviation (RMSD) between the MD predicted and the NMR observed chemical shifts is the best indicator of the agreement between an experimental structure and its theoretical model:^52,53^ their values are reported in Table 1 and the corresponding distributions in Figures S1–S3. They all evidence that, unsurprisingly, with respect to the starting NMR structure, the addition of dynamics improves the reproduction of chemical shifts of Cβ, hence improving the reliability of the spatial representation of the sidechains and of the solvent-accessible surface of the system. The secondary structures of the three proteins are well represented, as documented by the secondary chemical shifts (Figure S4). The Cα chemical shifts of WT and of E219K HuPrP are quite unaffected by the addition of dynamics to structure; hence, the experimentally obtained bundle represents in these cases a good representation of the overall structure of the proteins. In this regard, see the red and blue-striped histograms in Figures S1 and S2, which represent the overall RMSD of the MD-simulated structures and the NMR structure of WT HuPrP, respectively; they appear very close to each other, indicating that the dynamics reproduce the experimental chemical shifts of the WT and E219K structures. The reproduction of V210I mutant Cα chemical shifts, conversely, exhibits a significant improvement due to the addition of dynamics to experimental structures through MD simulations (see the lower RMSD of the red bar in Figure S3), thus suggesting that the standard bundle-based representation of the V210I NMR structure might be potentially improved. This improvement, together with the overall good reproduction of the available experimental data for the other structure, supports in the following the usage of this MD simulations approach^54^ as a reference for the structure and dynamics of the unbound HuPrP NMR constructs.

**Table 1:** Comparison Between the Cα and Cβ Chemical Shifts RMSD/ppm of MD-Simulated and NMR-Based HuPrP WT, E219K, and V210I

		Cα				Cβ			
		RMSD min value^a^	RMSD max value^b^	RMSD average^c^	RMSD start^d^	RMSD min. value	RMSD max. value	RMSD average	RMSD start
MD	WT	0.922	1.508	0.982	1.029	0.855	1.348	0.852	1.193
	E219K	1.106	1.670	1.151	1.211	0.924	1.485	0.952	1.139
	V210I	1.019	1.737	1.106	1.424	0.904	1.529	0.983	1.214
NMR	WT	0.993	1.058	0.989		1.107	1.193	1.113	
	E219K	1.094	1.228	1.124		1.153	1.255	1.136	
	V210I	1.424	1.257	1.319		1.058	1.214	1.092	

a Lowest value of the RMSD/ppm computed for a single structure over the whole distribution.

b Highest value of the RMSD/ppm computed for a single structure over the whole distribution.

c Average value of the RMSD/ppm computed over the whole distribution.

d Value of the RMSD/ppm computed for the starting structure used for MD simulations, that is, the lowest energy structure belonging to the NMR bundle.

The root mean square fluctuation (RMSF) of the Cα positions was monitored through the entire length of the MD simulations and computed over the concatenation of three distinct trajectories, that is, nominally the RMSF of Cα of a 3 μs trajectory for each HuPrP system. The analysis on *apo* HuPrP reveals larger conformational flexibility in the E219K and V210I relative to the WT. These regions were the β2−α2 loop (residues 160–175) and the α2−α3 loop region (residues 180–205), as obtained by the plot of the Cα root mean square fluctuation (RMSF) (Figure 2B). Single 1 μs RMSF replica for each protein system in the *apo* forms are shown in Figure S5. Notably, the 3 μs-RMSF trajectories of HuPrP-Nb484 complexes show a marked effect of the Nb in reducing the flexibility of the regions involved in the protein–protein interaction, that is, the segments from residues 123 to 125 and from 164 to 185 (Figure 2C). This confirms the ability of the Nanobody to trap the folding of flexible antigens present in both WT and mutant forms.

**Figure 3:**
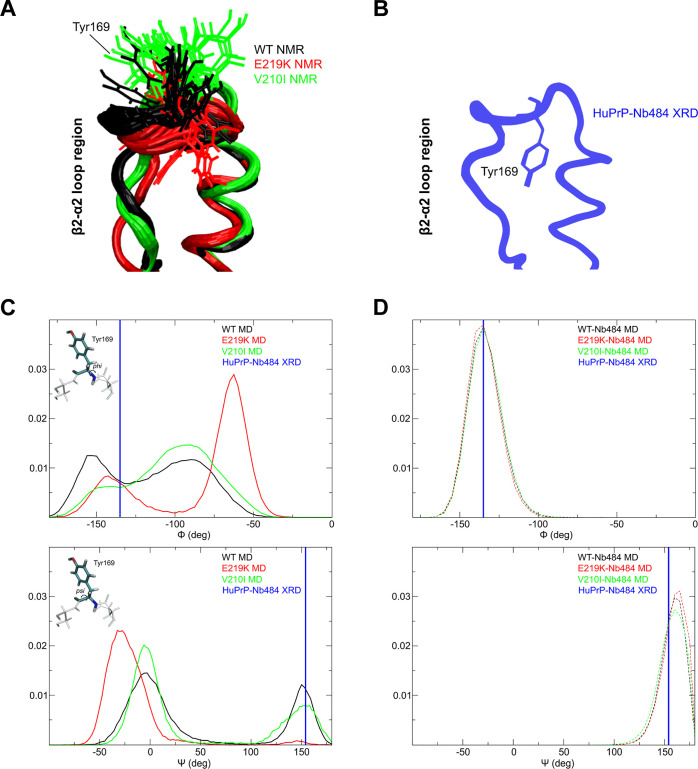
Local structural variation at the β2−α2 loop region. (A) Superposition of WT, E219K, and V210I NMR (A) and WT HuPrP-Nb484 (B) structures in the region encompassing the β2−α2 loop (residues 164–185). Conformation transitions of Tyr169 residue (black, red, and green for WT, E219K, and V210I, respectively) pointing outside (toward the solvent) in the NMR bundles (“open” conformation) or inside (“close” conformation) as observed in the WT HuPrP-Nb484 XRD structure. Ramachandran *phi* (upper panel) and *psi* (lower panel) dihedral angle trajectory distributions in MD *apo* (C) and *holo* (D) structures (black, green, and red for WT, V210I, and E219K, respectively) and in the Nb484-bound WT HuPrP-Nb484 XRD structure (blue bar).

The E219K polymorphism shows a higher flexibility degree within the β2−α2 loop, as is visible in both the RMSF and Tyr169 dihedral angle plots (Figures 2B and 3B). This feature was previously observed in the E219K NMR structure, and it is caused by the variation of the surface electrostatic potential due to the K219 substitution that affects the long-range contacts at the interface between the β2−α2 loop and the *C*-terminal end of the α3 helix.^44^ Besides the β2−α2 loop region, the V210I MD behavior shows increased flexibility also in the α2−α3 loop region, visible as a small spike in the RMSF plot (Figure 2B). Several specific salt bridges and hydrophobic long-range interactions in the helical interface play a crucial role in fold stabilization of this region in the WT HuPrP and their importance is supported by the observation that these interactions are absent also in other disease-linked HuPrP mutants, including V210I.^45,46,55,56^

### Structural Plasticity of the HuPrP β2−α2 Loop

As observed in different HuPrP NMR structures, the β2−α2 loop features local dynamic conformational changes. This segment shows π-stacking interactions between Phe175 and Tyr218, and long-range stabilizing hydrophobic interactions such as Y163–Y218, M166–Y218, Y169–F175, and F175–Y218.^56^ This hydrophobic organization in the β2−α2 loop is identical in the Nb484-bound WT HuPrP XRD structure.^34^ However, in this latter case, the Tyr169 side chain points toward the bulk of the protein showing a “close” conformation; conversely, in the NMR structures, such a side chain adopts a *trans* conformation, with the consequent exposure of the aromatic ring toward the solvent (Figure 3A). The dihedral angle distributions (*phi* and *psi* dihedral angles) confirm the “open” conformations of Tyr169 in all the MD structures. Here, some trajectories of all the three HuPrP systems also fall in proximity to the dihedral angles of Nb-bound WT HuPrP, suggesting that the Tyr169 “close” conformation may be preselected during the Nb484 binding, thus following a conformational selection mechanism (Figure 3B). Additionally, we have calculated the frequencies of the preferential Tyr169 dihedral angles in the three HuPrP and compared them with the crystallographic angles of the WT here used as a reference structure (Table S1). Here, it appears evident that WT and V210I Tyr169 dihedral angle populations display similar frequencies along the midline represented by the reference position of the XRD structure, while E219K *phi*/*psi* distributions are different. This is also evident in the plot for E219K Tyr169 dihedral angle distributions showing only the *phi* angle distributions overlapping with Nb-bound WT HuPrP (Figure 3B, upper panel) and in the Ramachandran plot for this selected residue (Figure S9). MD simulations support previous experimental observations that E219K HuPrP has higher flexibility in the β2−α2 loop that explores some trajectories competent for Nb binding. Conversely, in the *holo* forms, Tyr169 dihedral angles are stable in all the three HuPrP during the entire MD simulations and are clearly “trapped” by the Nb484 in a “close” conformation (Figure 3C).

### Conformational Variability between MD, NMR, and Nb484-Bound HuPrP Structures

The different conformational behaviors of WT, E219K and V210I HuPrP structures were assessed through the calculation of RMSD computed on Cα atoms (Table 2). MD simulations on the WT HuPrP display a backbone RMSD value around 1.9 Å with respect to the average structure of the NMR bundle and the XRD structure. This comparison already adds evidence on the conformational selection mechanism involving the recognition of Nb484 to HuPrP. Within the RMSD variations of the WT, the most perturbed regions are the ones comprised between residues 120 and 128 and the ones comprised between 163 and 173 (Figure 2B), that is, those that directly interact with the Nb484.

**Table 2:** Comparison Between the RMSD Cα (Å) of the HuPrP Structures Obtained by MD, NMR, and XRD

	WT		E219K		V210I	
	RMSD	σ_RMSD_	RMSD	σ_RMSD_	RMSD	σ_RMSD_
MD *versus* NMR structure	1.9	0.2	3.0	0.3	3.4	0.4
MD *versus* NMR structure without β2−α2 loop^a^	1.7	0.2	2.7	0.3	3.1	0.4
MD only	1.9	0.2	2.8	0.4	3.0	0.3
MD only without β2−α2 loop	1.6	0.3	2.7	0.3	3.0	0.4
MD *versus* HuPrP-Nb484 XRD	1.9	0.2	2.2	0.3	2.2	0.6
MD *versus* HuPrP-Nb484 XRD without β2−α2 loop	1.6	0.3	1.9	0.3	1.9	0.5

a Residues 163–173.

**Figure 4:**
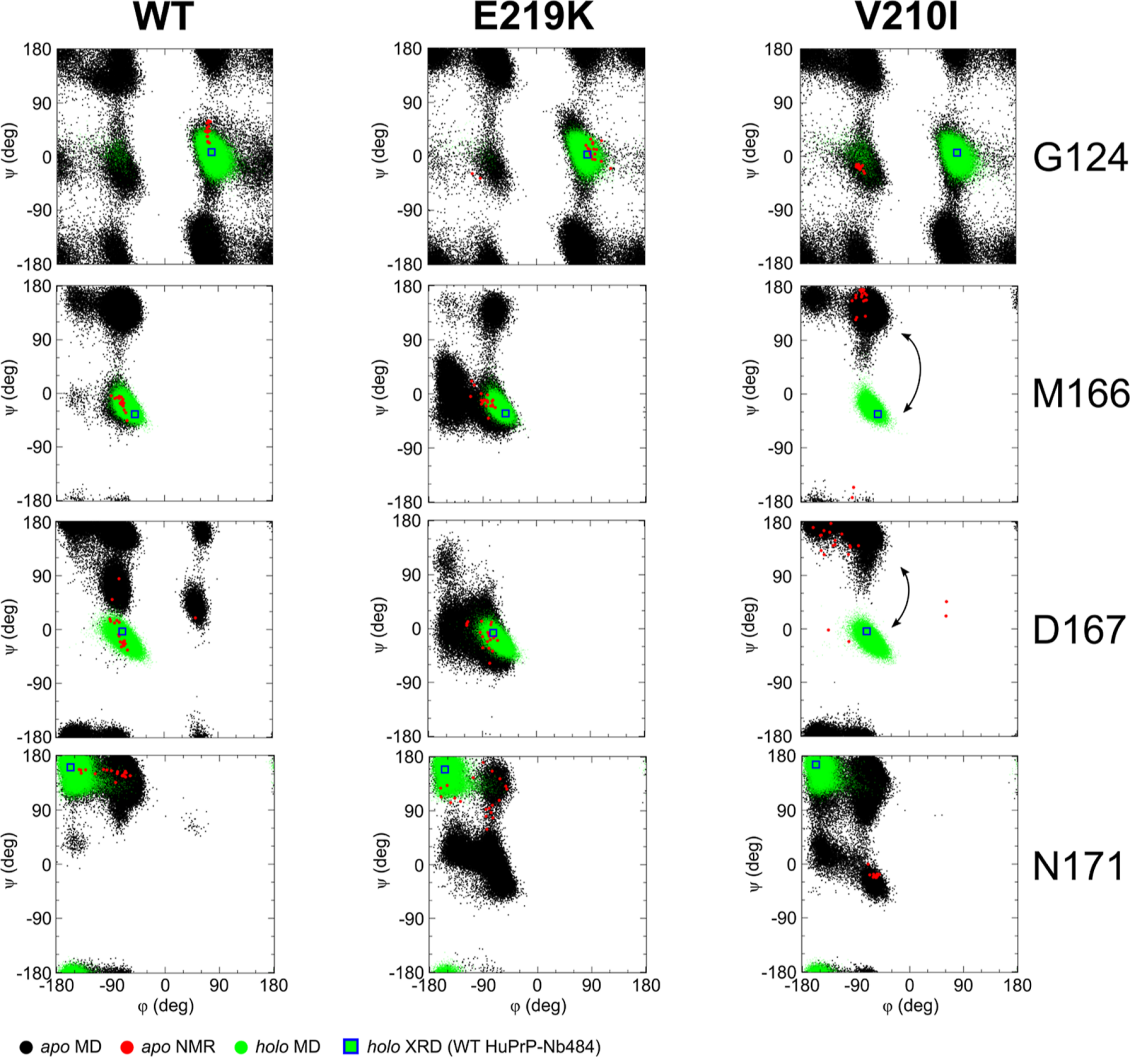
Ramachandran plots for selected residues of WT, E219K, and V210I HuPrP. The numbers of key residues involved in conformational changes (G124, M166, D167, and N171) are indicated on the right, while the HuPrP construct (WT, E219K, and V210I) is indicated on the top. In black dots, *phi*/*psi* pairs from MD snapshots (*apo* MD) are reported; in red dots, the ones extracted from the NMR structures in the deposited bundle (*apo* NMR); in green dots, the ones from simulated WT, E219K, and V210I HuPrP bound to Nb484 (*holo* MD); and in blue squares with green background, the ones extracted from the crystallographic structure of WT HuPrP bound to Nb484 (*holo* XRD). In the M166 and D167 panels, corresponding to the V210I mutant, the arrows highlight the arrangements of dihedral angles of these residues that occupy totally different positions in the Ramachandran space compared to the XRD structure. To better appreciate these arrangements, we show in Figure S6 the same Ramachandran plots without the green dots corresponding to *holo* MD.

To effectively represent the variations of dihedral angle positions of selected residues involved in the binding with Nb484, we used Ramachandran plots. Here, we show the *phi*/*psi* distributions extracted from the three *apo* HuPrP-MD, *holo* HuPrP-MD, the HuPrP-NMR, and WT HuPrP XRD structures (Figures 4 and S6). These residues, according to the analysis of dihedral dynamics, seem to be rearranged according to the angles that are mostly accessible to MD simulations as, for instance, in the case of residue Gly124. The crystallographic structure of the WT HuPrP bound to Nb484 shows that water molecules promote stability of the complex, especially in the β2−α2 loop region in proximity of residues from 167 to 169 (Figure S7), hence stabilizing their dynamics with respect to the free form in solution.^34^ In particular, the Asn171 sidechain (atom Oδ1) has a role in the stabilization via a direct contact with the Nb484 backbone (His177), as well as backbone interactions between Gly123 (on the HuPrP side) and Ile102 (on the Nb484). Interestingly, the Asn171 backbone is arranged in the XRD structure in a way that corresponds, in the Ramachandran plot, to a minor population of the *phi*/*psi* distribution obtained through MD simulations (Figure 4). This suggests that the interaction with Nb484 promotes in HuPrP WT a local configuration that allows better spatial accommodation of the Asn171 sidechain that, at the same time, requires distortion of the neighbor’s geometry, as shown by residues Asp167 (Figure 4) and Ser170 (Figure S8). Ramachandran plots for Tyr169 show dihedral dynamics that are conformationally accessible to Nb484 recognition, this is particularly evident in both WT and V210I and, to a lesser extent, in E219K as previously discussed (Figure S9).

The higher values and broader distributions of RMSD for E219K and V210I *apo* forms with respect to the WT HuPrP within their MD simulations reveal a more complex internal dynamics than in the WT, suggesting the presence of a larger set of structures that at room temperature are available for the interaction with molecular partners (Table S2).

**Figure 5:**
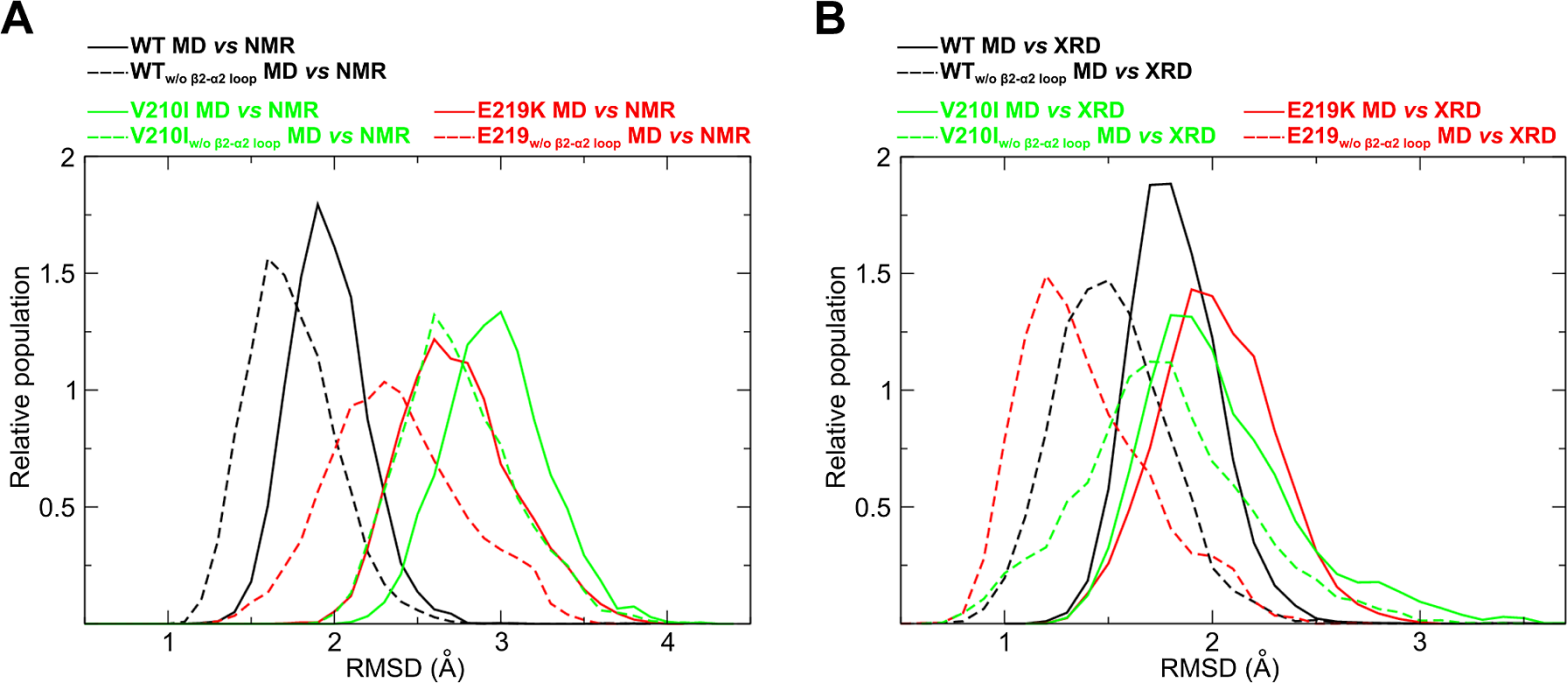
Mapping the β2−α2 loop conformational ensembles of WT, E219K, and V210I. (A) RMSD of the backbone of *apo* WT HuPrP (black), *apo* E219K (red), and *apo* V210I (green) computed on the backbone atoms with respect to corresponding NMR bundles. (B) RMSD of the backbone of *apo* WT HuPrP (black), *apo* E219K (red), and *apo* V210I (green) computed on the backbone atoms with respect to the XRD structure of the WT in complex with Nb484. The data with and without (w/o) the inclusion of the β2−α2 loop (residues 163–173) are reported as continuous and dashed lines, respectively.

With respect to experimental NMR structures, the distributions of RMSD values for the *apo* WT MD are quite small, whereas the ones of *apo* E219K MD and the *apo* V210I MD are broader and centred at around 2.5–3 Å, with a superimposition of small extent with the WT distributions for both of them (Figure 5A). The average values are in agreement with the retention of the overall protein folding, whereas the broadness of the distribution suggests a more complex dynamic that governs the recognition mechanisms of E219K and V210I. The source of this complexity can be highlighted by the same analysis performed with the exclusion of the β2−α2 loop segment (residues 163–173) (Figure 5A): if the backbone RMSD distribution average is only slightly reduced for the WT, indicating that the β2−α2 loop internal dynamics weakly affect the overall protein dynamics, the two mutants under investigation exhibit a different behavior. For E219K, the distribution becomes broader due to the enrichment in low-RMSD structure populations: the exclusion of the β2−α2 loop significantly increases the similarity with NMR structures, indicating that this loop is dynamically strongly affected by the single point mutations, despite the overall structures are populated by many structurally neighbor states. For V210I, such effect is not visible and the distribution of RMSD values is only slightly shifted while exhibiting the same broadness: the quite large distance from the experimental structures demonstrates the presence of a highly complex dynamics, as also indicated by the computation of chemical shifts values which are greatly improved (Figure S3) by integrating the experimental structure with molecular dynamics sampling.

In order to bridge the MD simulations and the crystallographic structure through the possibility to describe the molecular recognition features of HuPrP toward Nb484, we performed the same analysis of MD simulations relative to the structure of WT HuPrP bound to Nb484 (Figure 5B). The average RMSD value is ∼1.5 Å, which is comparable to the resolution of the experimental crystal structure. The exclusion of the β2−α2 loop decreases the average value of RMSD between the crystallographic pose of WT HuPrP-Nb484 and its ensemble of structures accessible in solutions according to MD simulations, supporting the idea of a conformational selection mechanism during the molecular recognition of Nb484. Despite the absence of an experimental crystallographic structure of the mutants in complex with Nb484, we performed the same analysis for them in order to inspect the potential existence of a conformational selection mechanism acting toward the Nb484. Interestingly, the RMSD distributions of both mutants are highly superimposable with the one of the WT, with an average value centred between 1.5 and 2 Å. Surprisingly, the exclusion of the β2−α2 loop led to a dramatic decrease of RMSD distribution modal value of E219K and a great superimposition with the one of the WT, strongly suggesting that the dynamics of E219K overall contains all the conformers able to recognize Nb484. V210I exhibits, even if less pronounced, the same trend: noteworthy, the broad RMSD values distribution in this case is highly superimposable to the one of the WT, still supporting a similar dynamics of recognition.

These data overall suggest that the ensembles of structures of all three HuPrP systems are very similar, with the β2−α2 loop dynamics being the only element of HuPrP heavily influenced in its dynamics by the presence of a single amino acid substitution, even far away from this loop itself. This leads to the suggestion of an overall mechanism of conformational selection acting in the three proteins, that is, they retain the overall fold while recognizing Nb484 during their interaction with it. A summary of the overall geometric backbone RMSD values within the MD-, NMR-, and X-ray-HuPrP systems here investigated is presented in Table 2.

Interestingly, if the global fold and dynamics of the free *apo* protein are crucial for the mode of action of this mechanism, the local configurational space of this loop in the WT and in the E219K is different at the level of single amino acids, displaying a distribution of *phi*/*psi* angle pairs generally broader in this region for the E219K in agreement with the commented RMSD distributions (see, for instance, residues Asp167 and Asn171) but displaying an opposite behavior with respect to the WT at the local level (Figure 4). The proposed methodology is able to identify the limit of the region where spatial arrangement during molecular recognition is still retained, that is, residue Met166.

In the crystallographic structure of the Nb484-bound WT HuPrP, the N terminus refolds back forming the β0 strand and displaying backbone interactions between Gly123 and Gly124 (on the HuPrP side) and E102 (on the Nb484 side) that seem again to stabilize the complex formation. Such interaction is favored by the intrinsic highly dynamic profile of the glycine residues that are able to deeply explore the conformational space and find the best way to accommodate the rearrangement of all the surrounding residues. Such highly flexible behavior of the HuPrP *N*-terminus is present also in E219K and V210I as clearly visible in the broad distribution of *phi*/*psi* angles of residue Gly124 (Figure 4). During the binding to Nb484, the palindromic motif conformers that are most complementary to some pre-existing ligand conformations can be selected by Nb484, and the equilibrium shifts toward these conformers to form the novel *N*-terminal β0−β1 fold.

Another stabilizing factor is the formation of a coupled interaction between residue 168 of the WT form and residues 108 and 109 of the Nb484, between residues 173 and 174 of the WT and residues 61 and 62 of the Nb484, together with Asp178 that forms a dual interaction with the Nb484 backbone via its sidechain carboxylic function (Figure S7). As mentioned, the β2−α2 loop exhibits an overall almost pure conformational selection Nb484-recognition mechanism for what concerns the WT and likely the E219K. For the V210I mutant, we observe that the *phi*/*psi* angle pairs of M166 and D167 occupy totally unpopulated or extremely sparsely populated regions of the Ramachandran space compared to the corresponding Ramachandran position of the WT Nb484-bound structure (Figure 4). Since the β2−α2 loop is an epitope for Nb484 recognition, we propose that the Nb484 will bind the V210I mutant through a synergic action of the two distinct mechanisms of conformational selection and induced fit. In the mutant, such a net dynamic behavior in this loop represents a net difference with respect to E219K and WT, hence identifying a likely different recognition mechanism for the CJD-related mutant.

Nb484 binds to WT HuPrP and mouse PrP with high affinity (*K*_d_ values of 9.5 and 50 nM, respectively).^33,34^ As previously mentioned, the intrinsic flexibility of the Nb484 CDR2 loop is able to recognize a disordered epitope, such as the β2−α2 loop, following the conformational selection recognition mechanism. It is possible to argue that such binding would occur also in the E219K and V210I HuPrP. To add more evidence to this hypothesis, we utilized the Molecular Mechanics-Poisson-Boltzmann/Surface Area (MM-PBSA) method^57^ for calculating protein–protein binding energies in WT-Nb484 MD, E219K–Nb484 MD, and V210I–Nb484 MD. Hence, MD simulations and MM-PBSA are widely employed in studying protein–protein and protein–ligand binding.^58−60^Table S4 summarizes the contributions from the van der Waals energy, electrostatic energy, polar solvation energy, a non-polar energy contribution approximated using solvent-accessible surface area (SASA), and finally the estimated binding energy in the three *holo* HuPrP systems. From the calculated results, it can be noted that the electrostatic energy values are lower in E219K. This is not surprising considering the effect of K219 on the electrostatic surface potential of the protein as observed in the NMR structure.^44^ The slightly higher binding energy observed for the E219K–Nb484 system reflects the effect of the increased flexibility observed in the β2−α2 loop epitope. Overall, MM-PBSA analysis supports the notion that the Nb484 is able to bind PrP from different species (i.e., human and mouse) and carries disease-linked mutations like E219K and V210I.

### Sampling the Motions of WT, E219K, and V210I HuPrP

Principal component analysis (PCA) investigates, by means of dimensionality reduction, the overall global motions of the protein during the MD simulations and allows comparison of the conformational space explored by HuPrP in the presence of the E219K polymorphism or the V210I mutation. It is well known that overall protein motions are described by the first few eigenvectors computed on the basis of the covariance matrix of the atomic fluctuations. For this reason, in this study, we selected the first 10 eigenvectors for the calculation of significant motions of the trajectories (Figure S10 represents the 10 eigenvalues in decreasing order vs corresponding eigenvector for WT, E219K, and V210I HuPrP).

**Figure 6:**
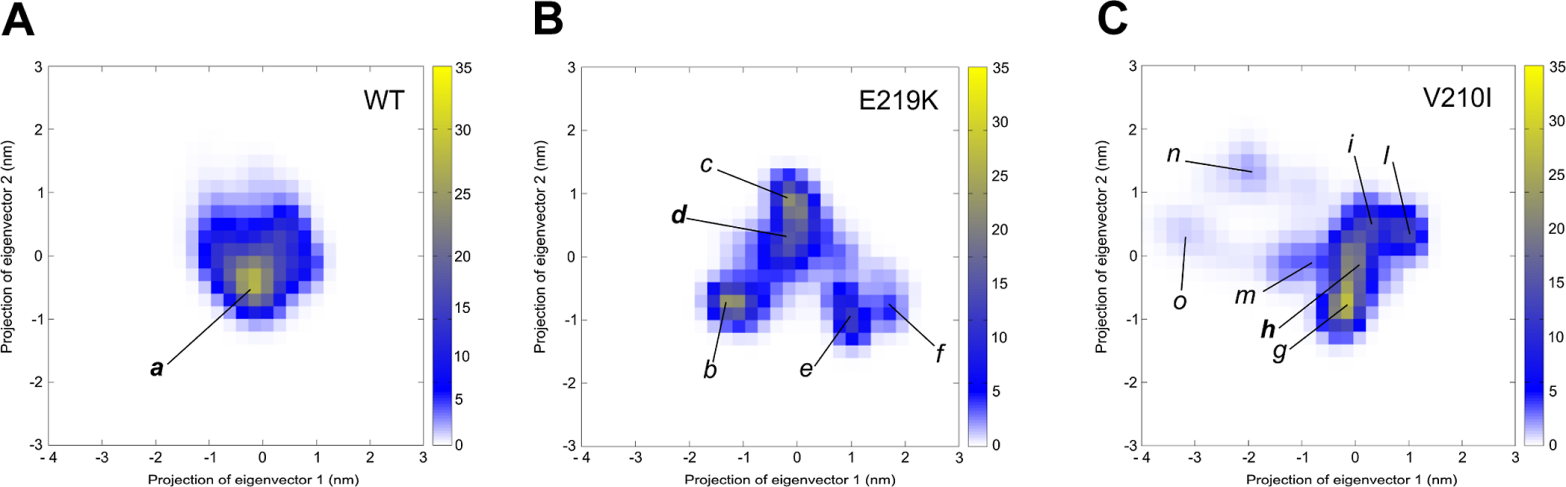
Principal component analyses (PCA). Projection of the motions of the proteins in phase space along the PC1 and PC2 is drawn for WT, E219K, and V210I in panels A, B, and C, respectively. Heatmap visualization of the distribution probability of the motion projections. The centroid populations (expressed in arbitrary values calibrated with a maximum height equal to 35 counts) are indicated with a letter code. In bold, the centroids showing the lower RMSD compared to the HuPrP-Nb484 structure (Table S3).

The results show that WT HuPrP has fewer motions as compared to E219K and V210I, indicating that these amino acid substitutions have different conformational variability effects on the protein structure as shown in the calculated 2D projection plots for WT, E219K, and V210I (Figure 6). In particular, the correlation graphs of the first two principal components (PC1 and PC2) showed that the WT displays a much more defined and stable cluster as compared to the E219K and V210I mutants. The distribution of PCAs for WT shows a concentration of the points in the middle area of the plot, indicating that there is limited heterogeneity in the motion of the protein (Figure 6A). Conversely, in both mutants, there is increased heterogeneity of the sampling. In E219K polymorphism, the motions cluster in overlapping regions sampling several configurations that make the protein more prone for conformational selection-based Nb484 recognition mechanisms (Figure 6B). In the CJD-linked V210I mutant, we clearly report net separation between the region of motion, indicating up to seven different centroids (here defined as representative conformers of each population identified on the basis of PCA), compatible with the experimental observation that V210I possesses β2−α2 loop dynamics in solution different from WT and E219K (Figure 6C). Furthermore, a distribution probability analysis within the PCA maps allowed to highlight the population of the centroids for each HuPrP system. Notably, the centroids indicated with the letter codes *a*, *d,* and *h* in the WT, E219K, and V210I plots, respectively, show global Cα RMSD values of around 1.4 Å with respect to the Nb-bound WT HuPrP (Table S3). This supports the hypothesis that the three HuPrP systems explore a configurational space similar to the Nb-bound state and add more evidence that the disordered epitopes of HuPrP are conformationally selected by Nb484 during the binding.

### Mechanisms of HuPrP–Nb484 Molecular Recognition

Our MD simulations on both *apo* and *holo* HuPrP forms may provide a picture of the early molecular mechanisms involved in the binding of the Nb484 to HuPrP. The MD-based *phi*/*psi* angle sequential distributions for the residues within the two HuPrP epitopes allow to detail the conformational selection mechanisms responsible for the binding. For the sake of clarity, we present here the analysis of the molecular recognition between WT HuPrP and the Nb484, for which both NMR and XRD data are available. Similar conclusions can be drawn for E219K and V210I HuPrP.

**Figure 7:**
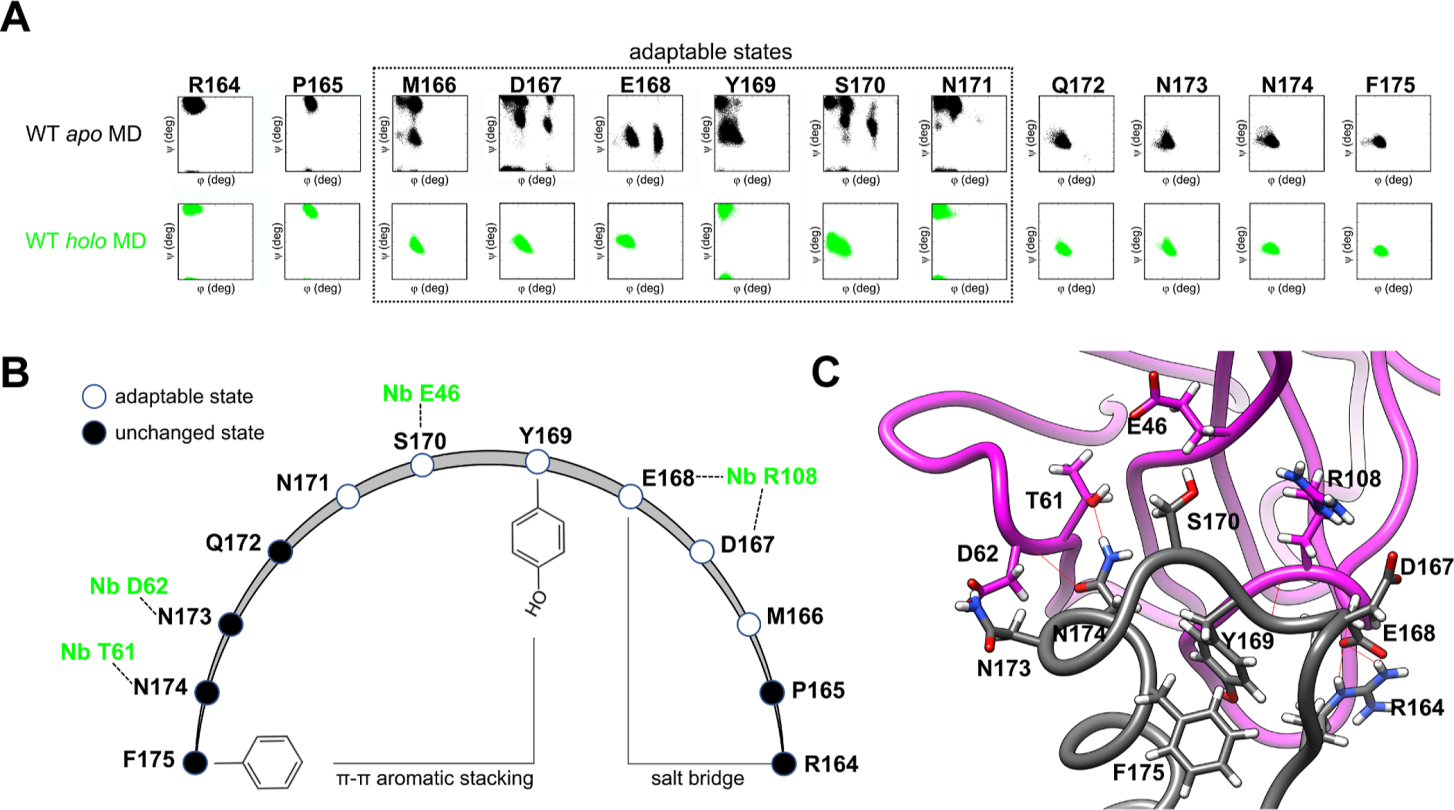
Mechanisms of WT HuPrP-Nb484 molecular recognition in the β2−α2 loop epitope region. (A) Ramachandran plots for selected residues within the β2−α2 loop. In black and green dots, the *phi*/*psi* pairs from MD snapshots from *apo* MD and *holo* MD, respectively. The dotted square indicates the residues involved in Nb484 interaction and subjected to adaptable changes during the conformational selection steps. (B) Schematic representation of the β2−α2 loop region, where black and white circles represent the residues involved in unchanged or adaptable states, respectively, during the binding with key residues (indicated in green) of Nb484. (C) Close overview of a MD snapshot from *holo* HuPrP with highlighted the residues involved in dynamic interactions (in gray and magenta color the HuPrP and Nb484, respectively).

In the β2−α2 loop, it emerges clearly that residues from M166 to N171 are “adaptable” and prone to conformational selection being their *phi*/*psi* distributions exploring the conformational spaces present in the Nb484-bound states (Figure 7A). N173 and N174 are located at the *N*-terminus of α2 and are trapped by D62 and T61, respectively, on the Nb484 side. In the loop S170, E168 and D167 are involved in H-bonding with the Nanobody and this lead to a structural stabilization of this segment and the formation of stable intra HuPrP interactions: π–π aromatic stacking between Y169 (in the “close” conformation) and F175; and a salt-bridge between E168 and R164 (Figure 7B,C). It is plausible that the electrostatic attraction between the HuPrP N173 and N174 drives one of the first steps of interaction with Nb484, and subsequently the other stabilizing interactions are formed within the β2−α2 loop.

As mentioned before, the segment corresponding to the β0−β1 strands in the XRD structure has an intrinsically highly dynamic profile in the *apo* form that makes it able to deeply explore the conformational space and find the best way to accommodate the structural rearrangements present in the *holo* state. Clearly, conformational selection mechanisms govern the interaction with Nb484. The peculiar hydrophobic signature of the so-called palindromic motif, together with the presence of hydrophobic residues (i.e., L100, I102, I105, and I105) in the CDR3 pocket favor the formation of van der Waals contacts between the two partners. This facilitates the formation of H-bonds within backbone residues in the segments 118–122 and 125–131, resulting in the formation of the β0−β1 hairpin. This novel secondary structure is largely facilitated by the presence of the conserved GGLGG sequence that confers flexibility and drives a structural change from an unfolded secondary structure in the *apo* to a loop connecting β0−β1 strands in the *holo* HuPrP (Figure S11).

## Conclusions

Understanding the fundamental principles of a protein binding to its protein-binding partner has received considerable attention due to the central role of protein/protein interactions in biology and the increasing demand for designing such specific interactions. The molecular mechanisms underlying the amyloid-based immunotherapy are complex, and deciphering the properties of amyloidogenic proteins responsible for these diseases is essential to obtain insights into antibody recognition of amyloid antigens. The molecular details of the recognition of disordered antigens by their cognate antibodies are less studied and known than folded protein antigens. Proteins with IDP domains may possess dominant conformations in the ensemble that can be stabilized by selected conformations by antibody binding.

It has been previously found that Nb484 binds with high affinity to WT HuPrP and mouse PrP, revealing unique structural features. The *N*-terminal palindromic motif adopts a stable and fully extended configuration to form a three-stranded antiparallel β-sheet, thus supporting the long standing hypothesis that the palindromic sequence AGAAAAGA mediates β-sheet formation in early stage of the PrP^C^ conversion to PrP^Sc^.^61^ Experimental data on this segment (residues 113–120) showed that it is largely disordered, and this clearly supports previous observations that antibody affinity to flexible amyloid epitopes is not affected by the epitope disorder.^50,62,63^

**Figure 8:**
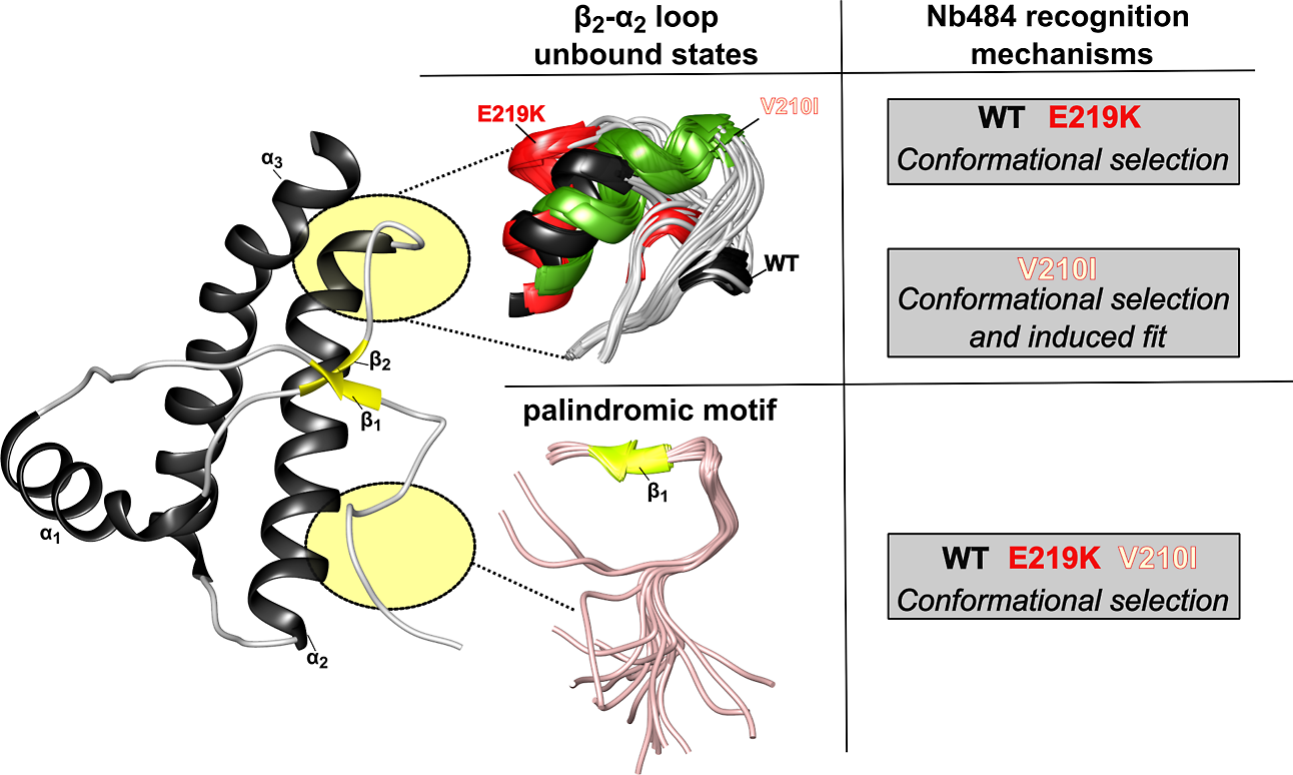
Proposed models for Nb484 recognition mechanisms in HuPrP carrying disease-related point mutations. On the left, the HuPrP β2−α2 loop and the palindromic motif (circled in yellow) are key sites for Nb484 interaction and highly flexible segments. The recognition of these antigens by Nb484 mainly follows the conformational selection model for the three HuPrP systems here investigated. In the CJD-linked V210I mutant, we also described a contribution of the induced fit mechanism for some residues within the loop.

In this study, we confirmed that the conformational selection model provides a realistic molecular recognition mechanism considering the conformational ensemble of the palindromic motif. Then, we have taken advantage of pre-existing WT, E219K, and V210I NMR structures to obtain a comprehensive and detailed picture of the conformational dynamics of the β2−α2 loop in these HuPrP structures. The conformational polymorphism of this loop in PrP^C^ plays a long debating role in the susceptibility and development of TSE.^42^ Using state-of-the-art MD methods in combination with NMR data, we designed the conformational landscapes of the β2−α2 loop to describe the Nb484-HuPrP recognition mechanisms in the WT and predict the behavior of this epitope in the E219K and V210I. Our analysis allowed us to describe that this loop is conformationally selected by Nb484 during the binding in all the three HuPrP systems. This is particularly evident in the WT and, to a lesser extent, in E219K, where several trajectories also explore the space competent for Nb binding. In the V210I mutant, we described a mixed contribution of conformational selection and induce fit that governs the recognition mechanisms since some residues clearly fall in trajectories competent for Nb484 recognition (i.e., Y169, N171, and S170) and others do not (i.e., M166 and D167); this seems due to the altered conformation of this loop as experimentally observed in previous NMR experiments.^45,46^ Notably, the PCA analysis relative to the V210I mutant shows that at least seven centroids may coexist in solution, indicating that this HuPrP possesses an increased propensity to explore different conformational spaces, which might also include pathological transitions to PrP^Sc^. A summary of the HuPrP-Nb484 recognition mechanisms is shown in Figure 8.

The first cryo-EM structure of the mammalian PrP^Sc^ fibrils provided key insights into the early events of prion conversion.^20^ These might involve the detachment of the β1−α1−β2 loop away from the α2 and α3 helices.^64^ Importantly, disease-related mutations are clustered almost in the folded part and may contribute to destabilize the native HuPrP conformation, promoting spontaneous prion conversion. Previous NMR studies on HuPrP carrying point mutations already reported high structural disorder of the β2−α2 loop and increased distance between this loop and α2−α3 helices. We and others interpreted these findings as an early event of protein unfolding that precedes the detachment of the β1−α1−β2 loop segment.^65−67^ Nb484 is able to recognize these highly dynamic and amyloid HuPrP antigens whose flexibility appears increased in both E219K polymorphism and in the CJD-linked V210 mutant. These observations underline the efficacy of this Nb to stabilize and trap the folding of aggregation-prone HuPrP mutants responsible for genetic human prion diseases. The selection of a Nanobody, such as Nb484, that preferentially binds flexible epitopes critical for TSE has expanded the repertoire of experimentally accessible states on PrP^C^. This is of key importance for structural and functional studies and for providing pharmacologic agents against prions with the potential to be targeted to specific cell types or tissue compartments.

## Methods

### Molecular Dynamics Simulations and Chemical Shift Calculations

The 105-amino acid HuPrP WT, E219K, and V210I NMR structures have been obtained from the Protein Data Bank, where they are deposited respectively with ID 2LSB (WT), 2LFT (E219K), and 2LEJ (V210I). The lowest energy structure of the bundle (i.e., the first frame) has been extracted for each HuPrP variant, and the region corresponding to residues 119–224 has been selected and parametrized using the AMBER99SB-ILDN^68^ force field in Gromacs 2020.7 suite.^69^ A parallelepipedal solvent box has been created around the protein, solvated with 3642 TIP3P^70^ water molecules, and the overall system charge has been balanced with counterions. After minimization with the steepest descent method (with convergence of the total force equal to 100 kJ mol^–1^ nm^–1^), the system has been equilibrated (with isotropic positional restraints on protein heavy atoms, *k* = 1000 kJ mol^–1^ nm^–2^) for 2 ns in the *NPT* ensemble with *p* = 1 atm and *T* = 300 K, then for 2 ns in the *NVT* ensemble at *T* = 300 K^71^ then we performed 3 replicas of 1 μs MD simulations (for a total of 3 μs) for each system in the *NVT* ensemble employing a timestep of 2 fs and constraining all covalent bond lengths with the LINCS algorithm.^72^ Experimental chemical shifts (δ_exp_) of WT, E219K, and V210I have been extracted and tabulated from the original papers,^44,46^ as reported in the Biological Magnetic Resonance Data Bank (BMRB): entries 18426 (WT), 17780 (E219K), and 17714 (V210I)). Chemical shifts have been predicted from single structures extracted from the trajectories of each simulation every 1 ns as also described in our previous studies^53,54^ using SPARTA+.^73^ The statistical homogeneity of the sampling during the simulations for each system has been verified by measuring the backbone RMSD of the region comprised between residues 124 and 222 (i.e., excluding the most flexible residues at the *N*- and *C*-termini) with respect to the starting structure. Moreover, the reliability and sampling homogeneity with respect to experimental data have been measured through the calculation of the RMSD of chemical shifts of Cα (for the backbone dynamics) and of Cβ (for the sidechain dynamics) of each trajectory. These values are reported in Table S2. The similarity of chemical shifts and backbone geometric RMSD values and of their standard deviations for each replica of each HuPrP variant under investigation supports the homogeneity of sampling and the reliability of simulations. Due to their statistical nature, all the analysis reported within the text are referred to an overall 3 μs dynamics obtained through the concatenation of the single replicas for each HuPrP variant.

The structure of the complex formed by WT HuPrP and Nb484 has been obtained from the Protein Data Bank, where it has been deposited with ID 4N9O. The structure is constituted by the residues 118–224 of HuPrP and by residues 1–124 of Nb484. Due to the unavailability of experimental structures of the HuPrP mutant complexes with Nb484, we have modeled them on the basis of the only available experimental complex structure, mutating the residues of interest, that is, replacing E219 with a lysine (E219K) and V210 with an isoleucine (V210I) using Pymol (The PyMOL Molecular Graphics System, Version 1.2r3pre, Schrödinger, LLC) in the contest of the whole complex.

The three complexes were parametrized according to the AMBER99SB-ILDN^68^ force field using Gromacs 2020.7.^69^ A parallelepipedal solvent box has been created around the protein, solvated with 15218 TIP3P^70^ water molecules, and the overall system charge has been balanced with counterions. After minimization with the steepest descent method (with convergence of the total force equal to 100 kJ mol^–1^ nm^–1^), the system has been equilibrated (with isotropic positional restraints on protein heavy atoms, *k* = 1000 kJ mol^–1^ nm^–2^) for 2 ns in the *NPT* ensemble with *p* = 1 atm and *T* = 300 K, then for 2 ns in the *NVT* ensemble at *T* = 300 K^71^ then we performed 3 replicas of 1 μs MD simulations (for a total of 3 μs) for each system in the *NVT* ensemble employing a timestep of 2 fs and constraining all covalent bond lengths with the LINCS algorithm.^72^ The similarity of backbone geometric RMSD values and of their standard deviations for each replica of each HuPrP variant under investigation supports the homogeneity of sampling and the reliability of simulations. Due to their statistical nature, all the analysis reported within the text are referred to an overall 3 μs dynamics obtained through the concatenation of the single replicas for each HuPrP variant.

The Molecular Mechanics Poisson–Boltzmann surface area (MM-PBSA) analysis of the trajectory of the three complexes has been performed using the SASA-based (solvent accessible surface area) APBS^74^ based *g_mmpbsa* tool^75^ for directly accessing the GROMACS 2020.7 trajectories alongside the original binary files used for performing the simulation. The original trajectories have been sampled in order to collect 300 frames over the whole 3 μs concatenated trajectory for each complex (i.e., 1 frame/10 ns), leading to an average value of each term of the MM-PBSA analysis together with its standard deviation (reported in Table S4). As suggested by the authors of the *g_mmpbsa* tool, ε_solute_ and ε_solvent_ have been assumed to be 2 and 80, respectively; the salt concentration has been set up at 150 mM; a grid resolution of 0.5 Å has been adopted; the solvent radius has been assumed to be 1.4 Å.

All the analysis reported in the text (root mean square deviation, root mean square fluctuations, dihedral angles, and principal component analysis) has been performed using GROMACS 2020.7 (respectively: *gmx rms*, *gmx RMSF*, *gmx chi*, *gmx covar*/*gmx anaeig*). The chemical shifts analysis has been performed by means of Bash/Awk in-house scripts. The graphs have been realized using Xmgrace and Gnuplot. The visual inspection of trajectories and the molecular graphics images have been realized using VMD 1.9.3^76^ and UCSF Chimera 1.16.^77^

## Data and Software Availability

Input files, initial and last conformations, and representative structures of intermediates for each prion protein system and MD simulations are publicly accessible and distributed under an open source license via GitHub at the following web addresses: https://github.com/luca-mollica/JCIM_HuPrP_08.2022 (*apo*), https://github.com/luca-mollica/JCIM_1strev_HuPrP_11.2022 (*holo*). Available data include: original PDB deposited for each PrP variant, starting structure of the MD simulations, protein only binary trajectorie (3 replicates of 1 μs) for each system analyzed in the manuscript (WT, E219K, V210I, *holo* and *apo* forms: the details are reported in the main text). Binary trajectories, including water and counterions, are available upon request. The SPARTA+ predicted chemical shifts (i.e., 3000 files for each system) are available upon request.

## Supporting Information Available

Details on preferential *phi*/*psi* distributions of Tyr169 in the HuPrP *apo* forms; RMSD values for the three replicas of each simulated HuPrP system; RMSD values of the PCA centroids structures; RMSD of chemical shifts distribution for Cα and Cβ and secondary chemical shifts differences for WT, E219K, and V210I HuPrP; RMSF plots of *apo* HuPrP showing single 1 μs RMSF replica for each protein systems; details on interactions at the interface of the β2−α2 loop and Nb484 in two WT HuPrP crystal structures solved in previous studies; and mechanisms of WT HuPrP-Nb484 molecular recognition in the palindromic motif epitope region (PDF)
