# Supplementary material for: Recognition Mechanisms between a Nanobody and Disordered Epitopes of the Human Prion Protein: An Integrative Molecular Dynamics Study
Source: J Chem Inf Model. 2022 Dec 29;63(2):531–45. doi: 10.1021/acs.jcim.2c01062 (PMC9875307; doi:10.1021/acs.jcim.2c01062)
Supplement: Supplementary file 1 — ci2c01062_si_001.pdf [file ci2c01062_si_001.pdf]

Supporting Information

Recognition mechanisms between a Nanobody and disordered epitopes of the human prion protein: an integrative molecular dynamics study

Luca Mollica[1*] and Gabriele Giachin[2*]

[1] Department of Medical Biotechnology and Translational Medicine, University of Milan, 20090 Segrate, Milan, Italy.

[2] Department of Chemical Sciences (DiSC), University of Padua, 35131 Padova, Italy.

* corresponding authors: luca.mollica@unimi.it and gabriele.giachin@unipd.it

Supporting Table S1. Preferential *phi*/*psi* distributions of Tyr169 in the HuPrP *apo* forms.

Angle	Structure	Leftmost peak (± 20°)*	Population	Rightest peak (± 20°)*	Population
phi Tyr169	WT	-153	30 %	-90	35 %
	E219K	-143	25 %	-70	56 %
	V210I	-143	20 %	-63	23 %
psi Tyr169	WT	0	45 %	153	27 %
	E219K	-30	60 %	152	2 %
	V210I	0	50 %	153	22 %

*: the evaluation of the percentages of the different populations has been performed counting the number of frames corresponding to a region centered on modes with a 60 degrees (± 30 degrees) span, considering this value reasonable for populating minimum free energy minima as a function of *phi/psi* angles.

Supporting Table S2. Geometric backbone (Å) and Chemical shifts (ppm) in RMSD values for the three replicas of each simulated HuPrP system, i.e., in the *apo* (upper part) and *holo* (lower part) forms.

	WT			E219K			V210I		
	Run 1#	Run 2#	Run 3#	Run 1#	Run 2#	Run 3#	Run 1#	Run 2#	Run 3#
**RMSD Cα (Å)***	1.9 ± 0.2	2.0 ± 0.2	1.8 ± 0.2	2.7 ± 0.3	2.9 ± 0.4	2.6 ± 0.2	3.1 ± 0.3	3.1 ± 0.3	2.9 ± 0.3
**RMSD $C\alpha_{merged}$****	1.9 ± 0.2 Å			2.8 ± 0.4 Å			3.0 ± 0.3 Å		
**RMSD δ(Cα) (ppm)**	0.977	1.007	1.018	1.150	1.153	1.213	1.100	1.159	1.085
**RMSD $\delta(C\alpha)_{merged}$****	0.982 ppm			1.151 ppm			1.106 ppm		
**RMSD δ(Cβ) (ppm)**	0.877	0.881	0.889	0.935	0.978	1.043	0.961	1.047	0.989
**RMSD $\delta(C\beta)_{merged}$****	0.852 ppm			0.952 ppm			0.983 ppm		

	WT			E219K			V210I		
	Run 1#	Run 2#	Run 3#	Run 1#	Run 2#	Run 3#	Run 1#	Run 2#	Run 3#
**RMSD Cα (Å)*****	1.9 ± 0.3	1.5 ± 0.3	1.9 ± 0.5	1.8 ± 0.3	1.7 ± 0.5	1.3 ± 0.2	1.4 ± 0.2	1.5 ± 0.2	1.6 ± 0.3
**RMSD $C\alpha_{merged}$****	1.8 ± 0.4 Å			1.7 ± 0.4 Å			1.5 ± 0.3 Å		

#: 1 μs of simulation for each run

*: geometric backbone RMSD values have been computed with respect to the starting structure used for the simulations, i.e., the lowest energy frame extracted from the corresponding NMR bundles of structures.

**: the merged values represent the RMSD values computed for all the frames extracted from the three replicas.

***: geometric backbone RMSD values have been computed with respect to the starting structure/template used for the simulations, i.e., the deposited crystal structure of HuPrP-NB complex (PDB ID: 4N9O).

Supporting Table S3. Average backbone RMSD values of the PCA centroids structures.

Centroid	RMSD $_{\text{Nb-bound HuPrP}}$ (Cα) WT (Å)	E219K (Å)	V210I (Å)
a	1.48 ± 0.11		
b		1.74 ± 0.11	
c		1.61 ± 0.10	
d		1.36 ± 0.05	
e		1.94 ± 0.15	
f		2.38 ± 0.14	
g			1.51 ± 0.09
h			1.36 ± 0.06
i			1.89 ± 0.10
l			1.55 ± 0.10
m			1.38 ± 0.11
n			2.61 ± 0.20
o			2.08 ± 0.15
Whole PCA RMSD$_{\text{Nb-bound HuPrP}}$	1.48 ± 0.21	1.66 ± 0.26	1.56 ± 0.26

Supporting Table S4. MM-PBSA terms calculated for the three MD HuPrP-Nb484 systems

	WT<br>HuPrP-Nb484 MD	V210I<br>HuPrP-Nb484 MD	E219K<br>HuPrP-Nb484 MD
**van der Waals energy (kJ/mol)**	-295.72 ± 1.65	-297.90 ± 2.12	-292.20 ± 1.92
**Electrostatic energy (kJ/mol)**	-910.68 ± 8.03	-747.26 ± 9.07	-929.04 ± 8.71
**Polar solvation energy (kJ/mol)**	652.85 ± 5.92	714 ± 10.4	707.22 ± 11.32
**SASA energy (kJ/mol)**	-32.33 ± 0.22	-32.77 ± 0.25	-32.25 ± 0.22
**Binding energy (kJ/mol)**	-588.95 ± 4.83	-363.38 ± 7.84	-546.25 ± 8.4

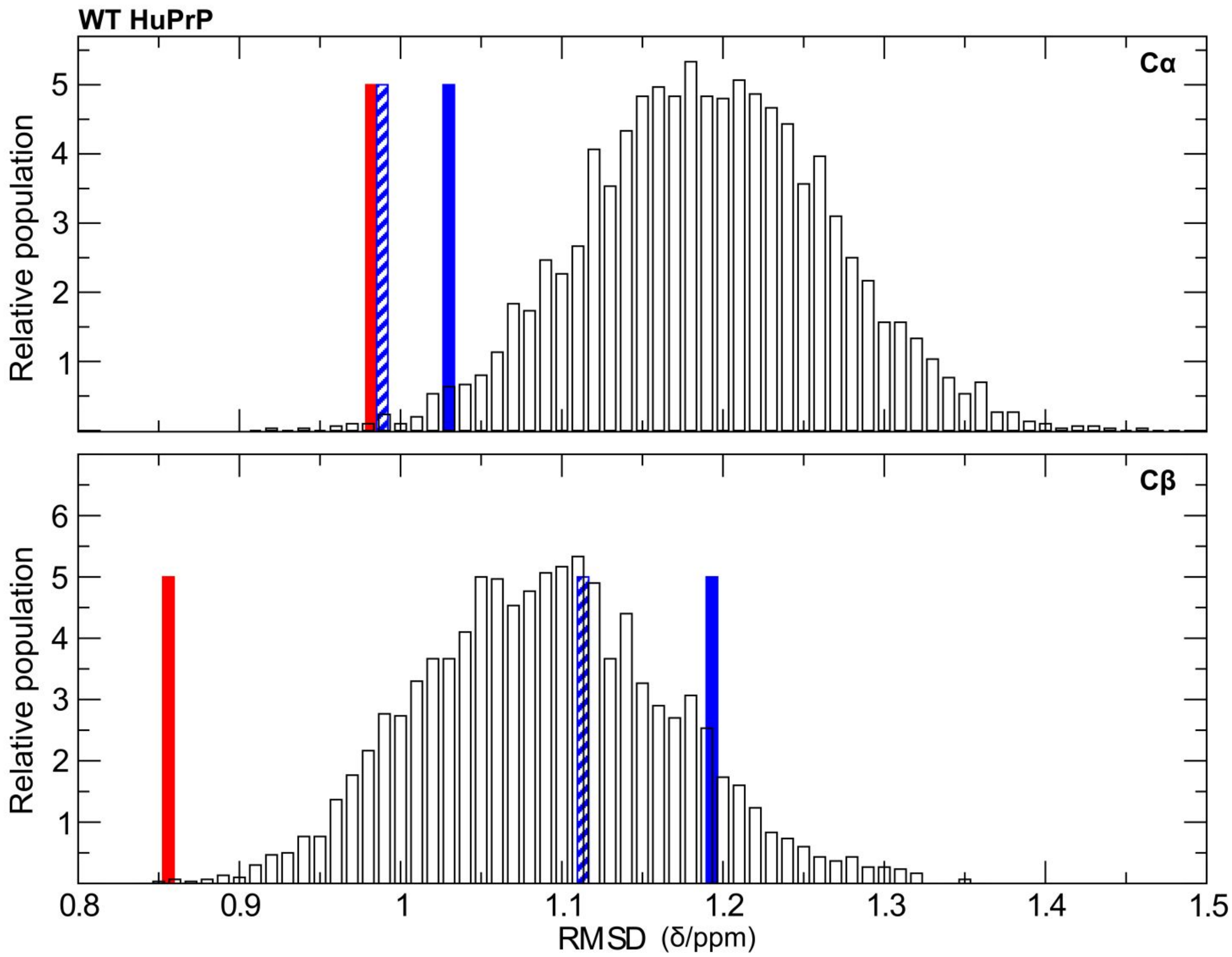

Figure S1. RMSD of chemical shifts distribution for Cα (*upper panel*) and Cβ (*lower panel*) for WT HuPrP. Red, blue and blue-striped histograms represent, respectively, the overall RMSD of the MD-simulated structures (frames), Nb484-bound structure and NMR structure of WT HuPrP, respectively.

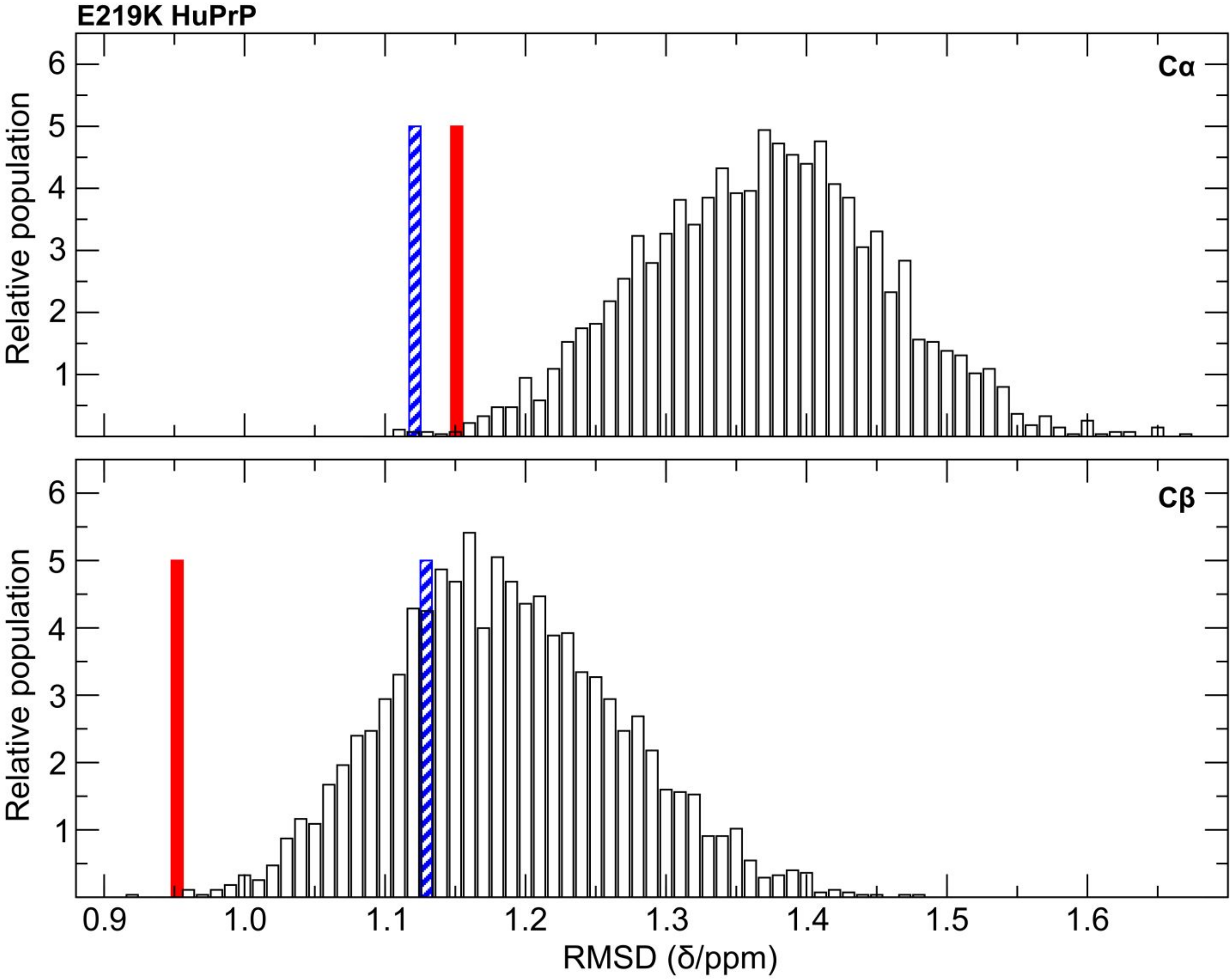

Figure S2. RMSD of chemical shifts distribution for Cα (*upper panel*) and Cβ (*lower panel*) for E219K HuPrP. Red and blue-striped histograms represent the overall RMSD of the MD-simulated structures (frames), NMR HuPrP E219K structures, respectively.

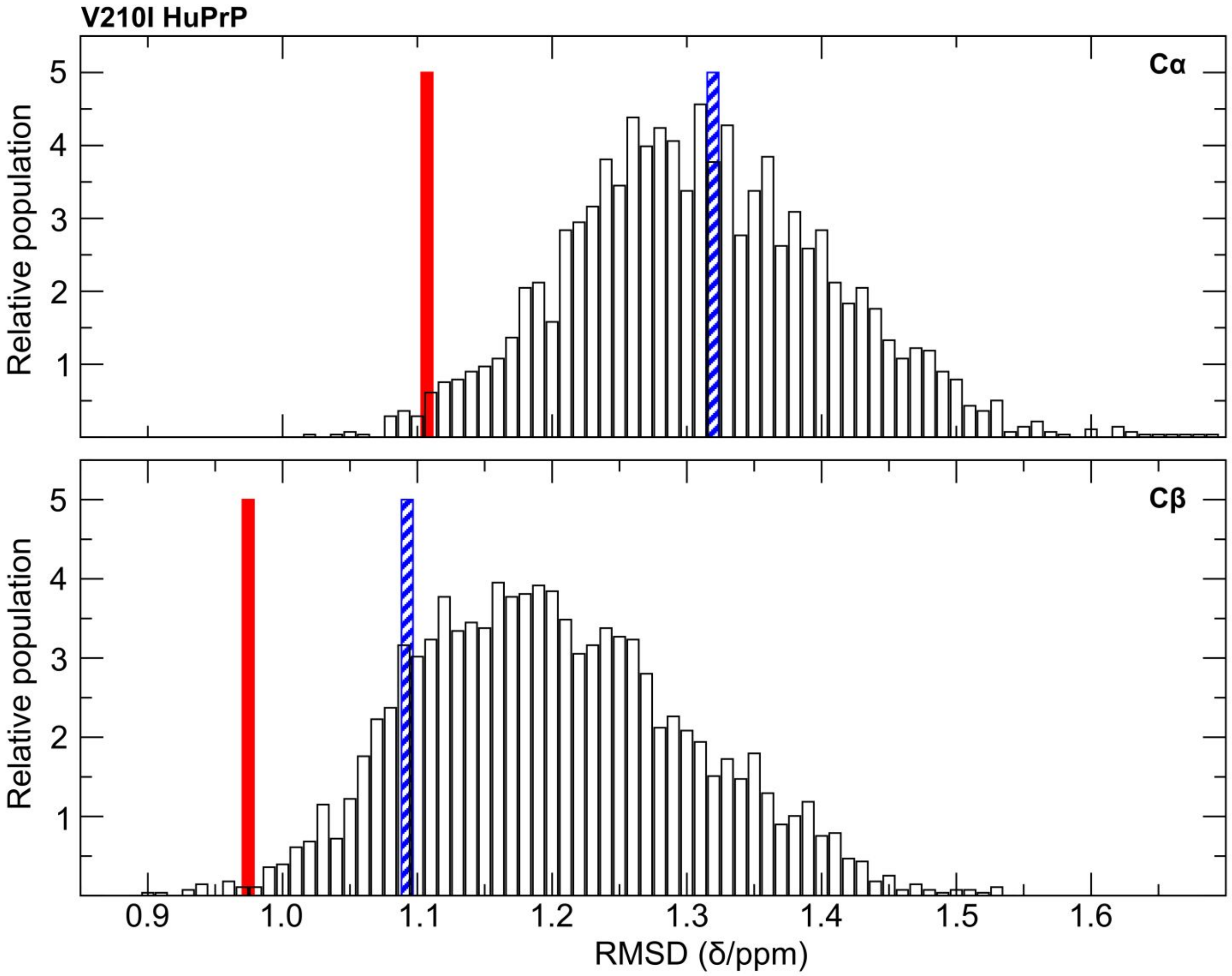

Figure S3. RMSD of chemical shifts distribution for Cα (*upper panel*) and Cβ (*lower panel*) for V210I HuPrP. Red and blue-striped histograms represent the overall RMSD of the MD-simulated structures (frames), NMR HuPrP V210I structures, respectively.

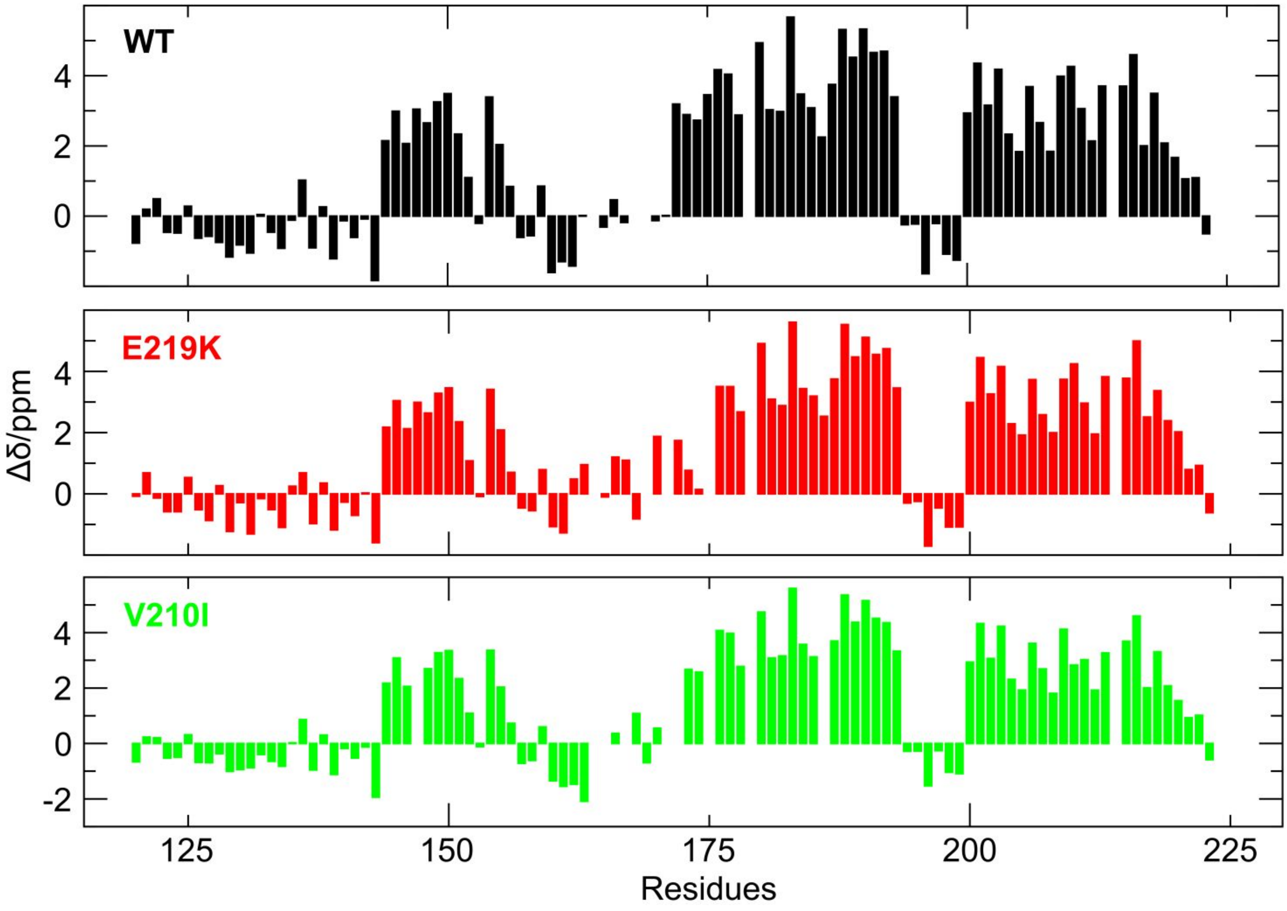

Figure S4. Secondary chemical shifts differences (δ_{NMR}-$\delta_{MD\ calculated}$) for the WT (black), E219K (red) and V210I (green) HuPrP.

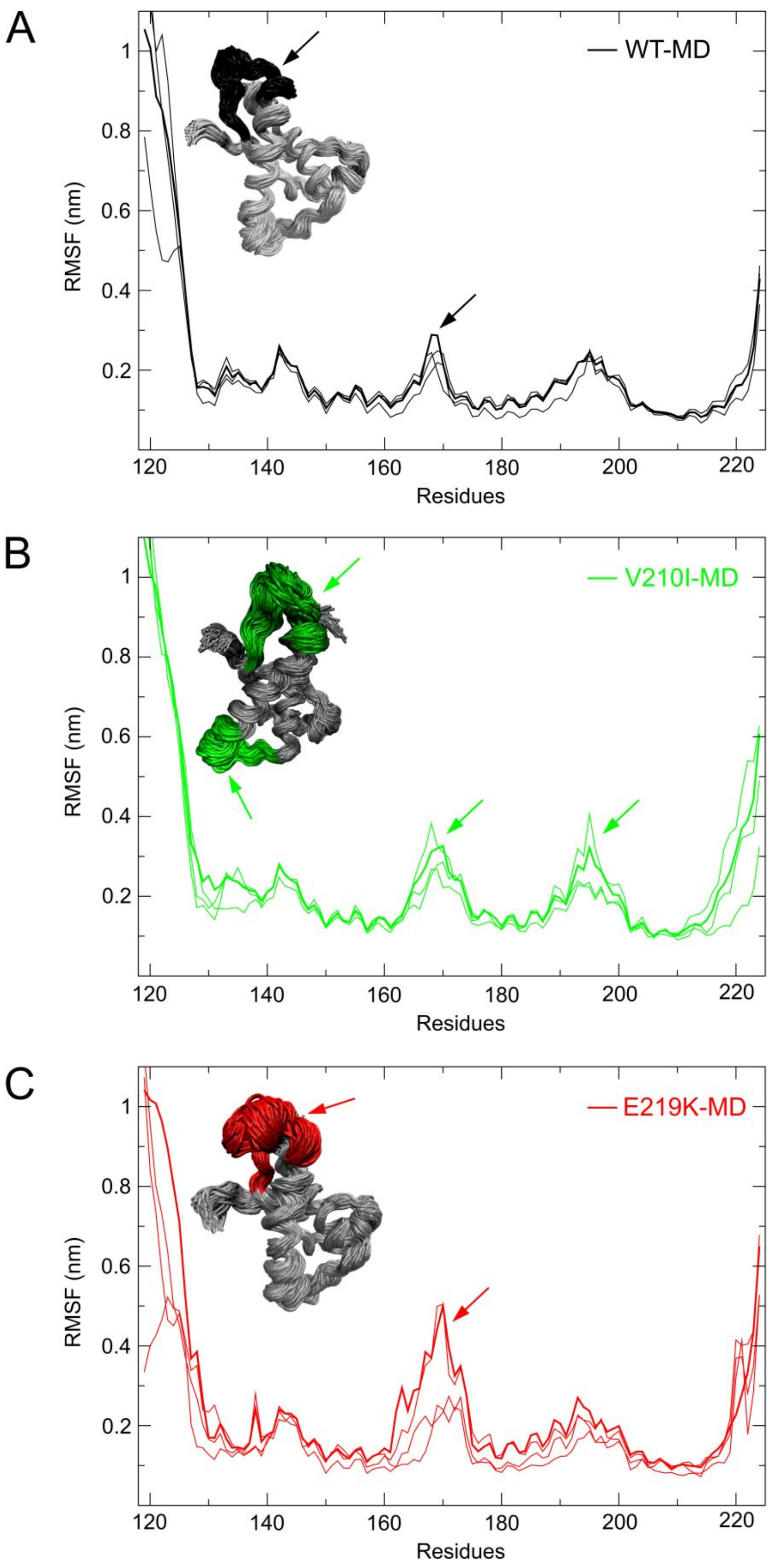

Figure S5. Root mean square fluctuations (RMSF) of unbound HuPrP. Single 1 µs RMSF replica (n = 3) for each protein systems are shown in black (for WT), green (for V210I) and red (E219K) in panel A, B and C, respectively. The thicker line represents the RMSF computed over the concatenation of three distinct trajectories (see **Figure 2 B**). The regions that showed higher flexibility are highlighted with a different colour and arrows in the MD structures of WT and mutants HuPrP (see in the inset).

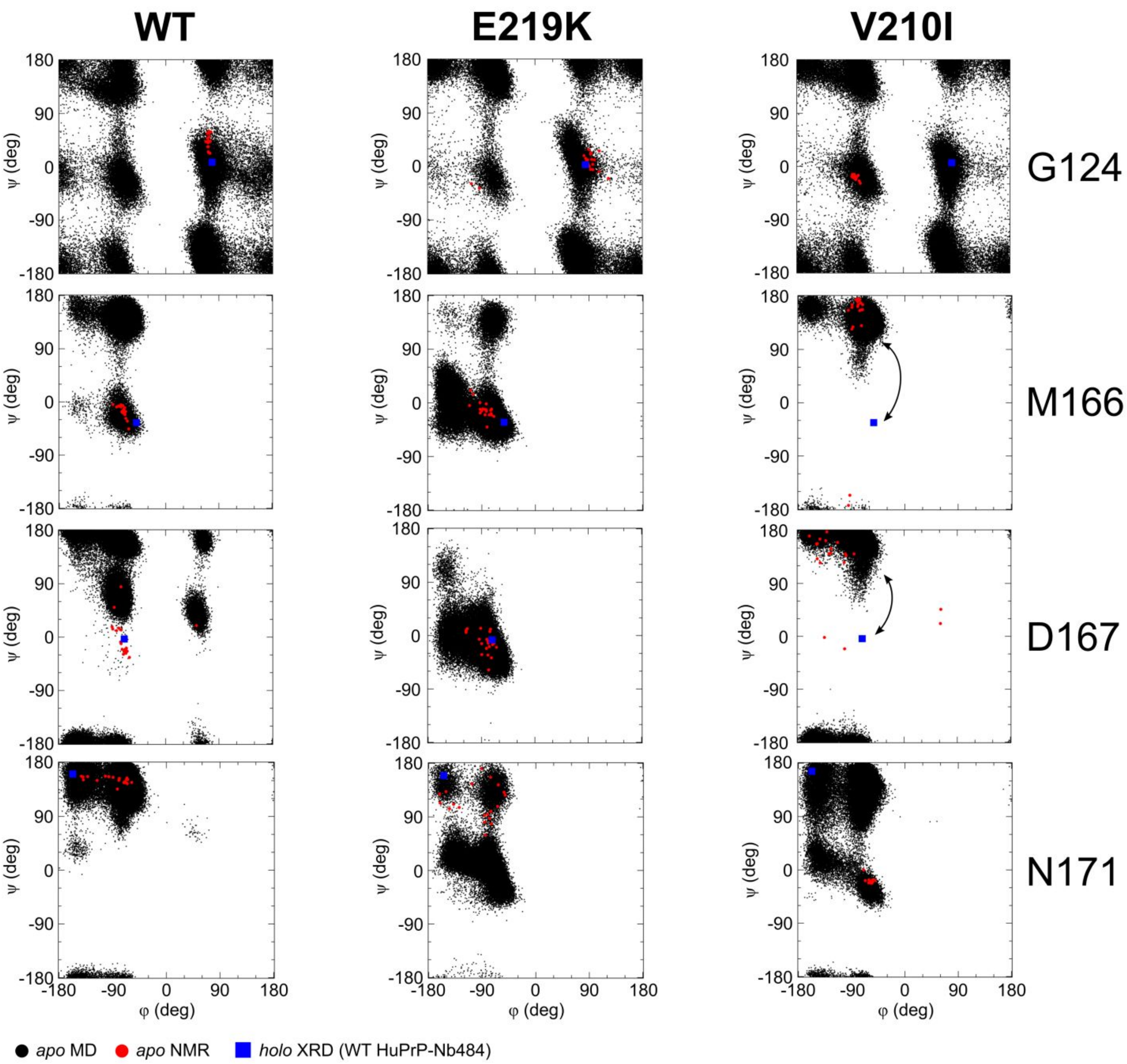

Figure S6. Ramachandran plots for selected residues of WT, E219K and V210I HuPrP. The numbers of key residues involved in conformational changes (G124, M166, D167 and N171) are indicated on the right while the HuPrP construct (WT, E219K and V210I) is indicated on the top. In black dots *phi*/*psi* pairs from MD snapshots (*apo* MD) are reported, in red dots the ones extracted from the NMR structures in the deposited bundle (*apo* NMR) and in blue squares the ones extracted from the crystallographic structure of WT HuPrP bound to Nb484 (*holo* XRD). In the M166 and D167 panels corresponding to V210I mutant, the arrows highlight the arrangements of dihedral angels of these residues that occupy totally different positions in the Ramachandran space compared to the XRD structure.

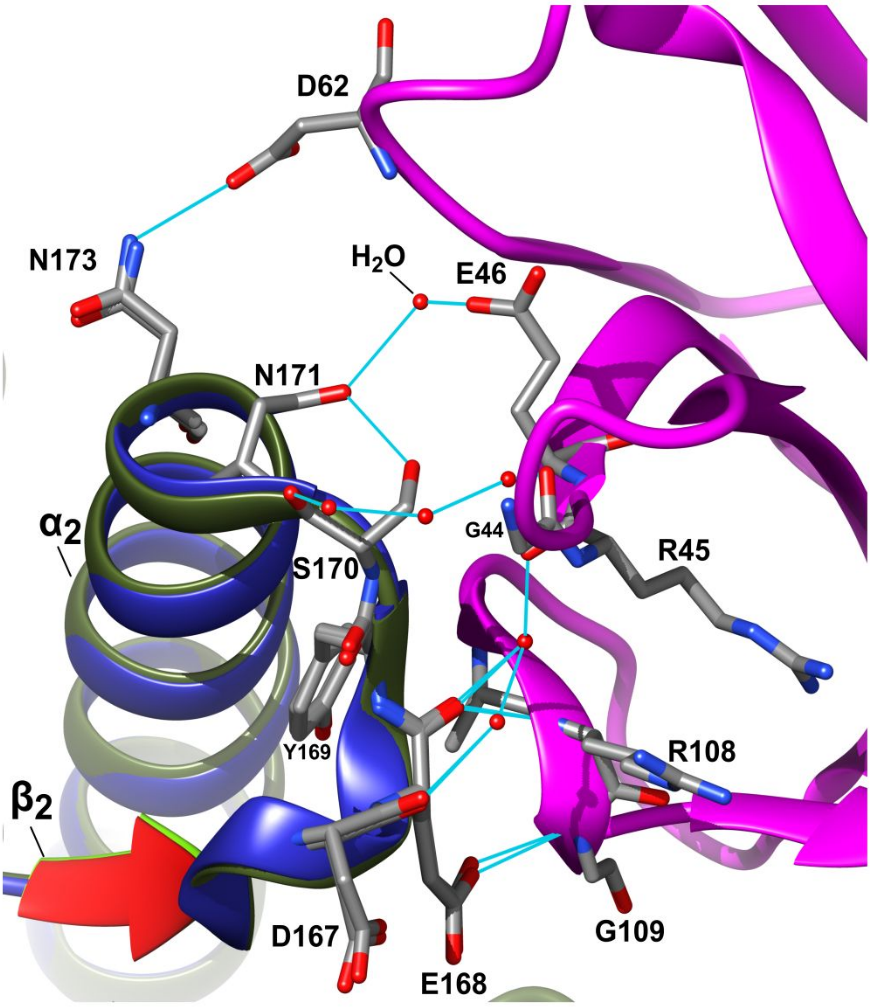

Figure S7. Interactions at the interface of the β2-α2 loop and Nb484 in two WT HuPrP crystal structures solved in previous studies: in green the PDB ID 4kml and in blue the PDB ID 4n9o. Nb484 is colored in magenta. Water molecules (in red) mediate protein-protein interaction and contribute to complex stability.

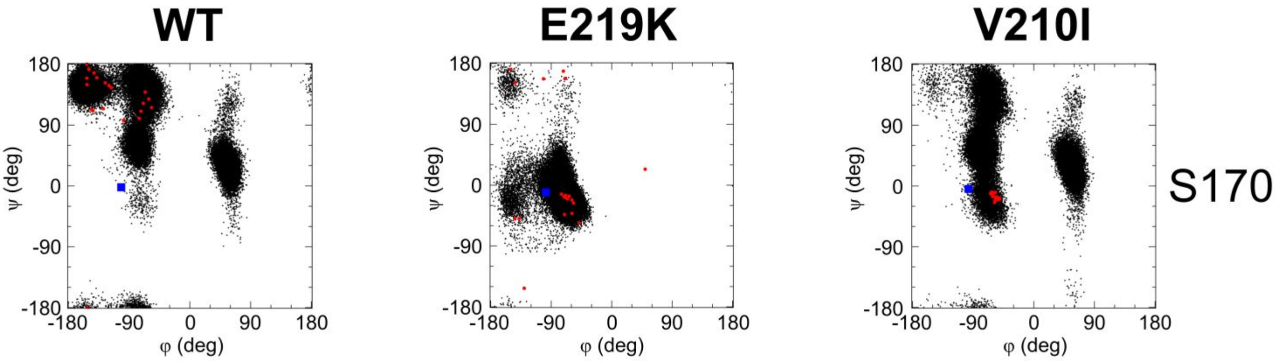

Figure S8. Ramachandran plots for residue Ser170 of WT, E219K and V210I HuPrP. In black dots *phi*/*psi* pairs from MD snapshots are reported, in red the ones extracted from the NMR structures in the deposited bundle, in blue the one extracted from the crystallographic structure of WT HuPrP bound to Nb484. For the sake of clarity, here, we omitted the *phi*/*psi* pairs from MD snapshots of WT, E219K and V210I bound to Nb484 (*holo* MD).

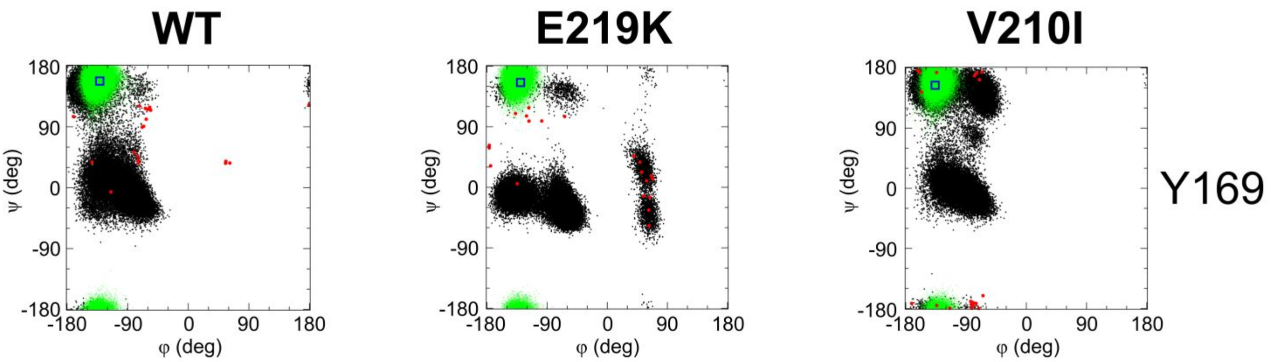

Figure S9. Ramachandran plots for residue Tyr169 of WT, E219K and V210I HuPrP. In black dots *phi*/*psi* pairs from MD snapshots are reported, in red the ones extracted from the NMR structures in the deposited bundle, in green dots the ones from simulated WT, E219K and V210I HuPrP bound to Nb484 and in blue squares with green background the one extracted from the crystallographic structure of WT HuPrP bound to Nb484.

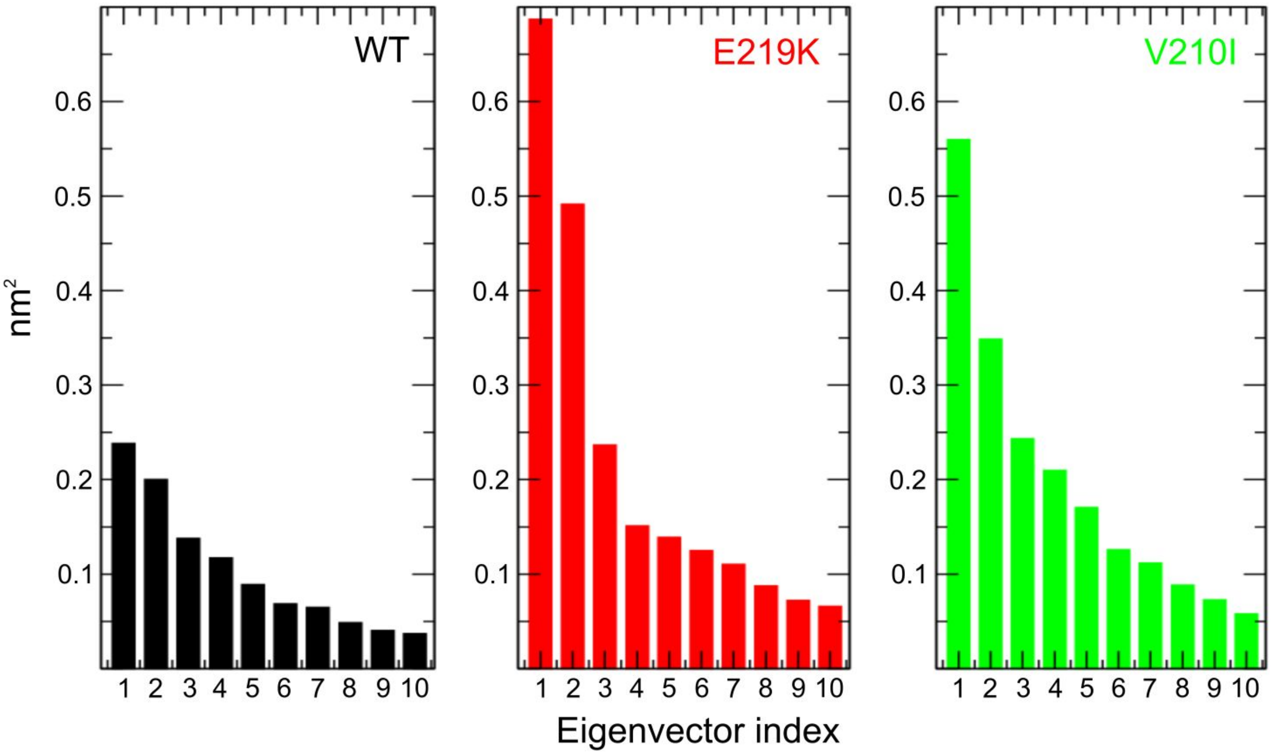

Figure S10. Principal component analysis of WT *versus* E219K polymorphism and V210I mutant. Plots of eigenvalues *versus* eigenvector index are shown.

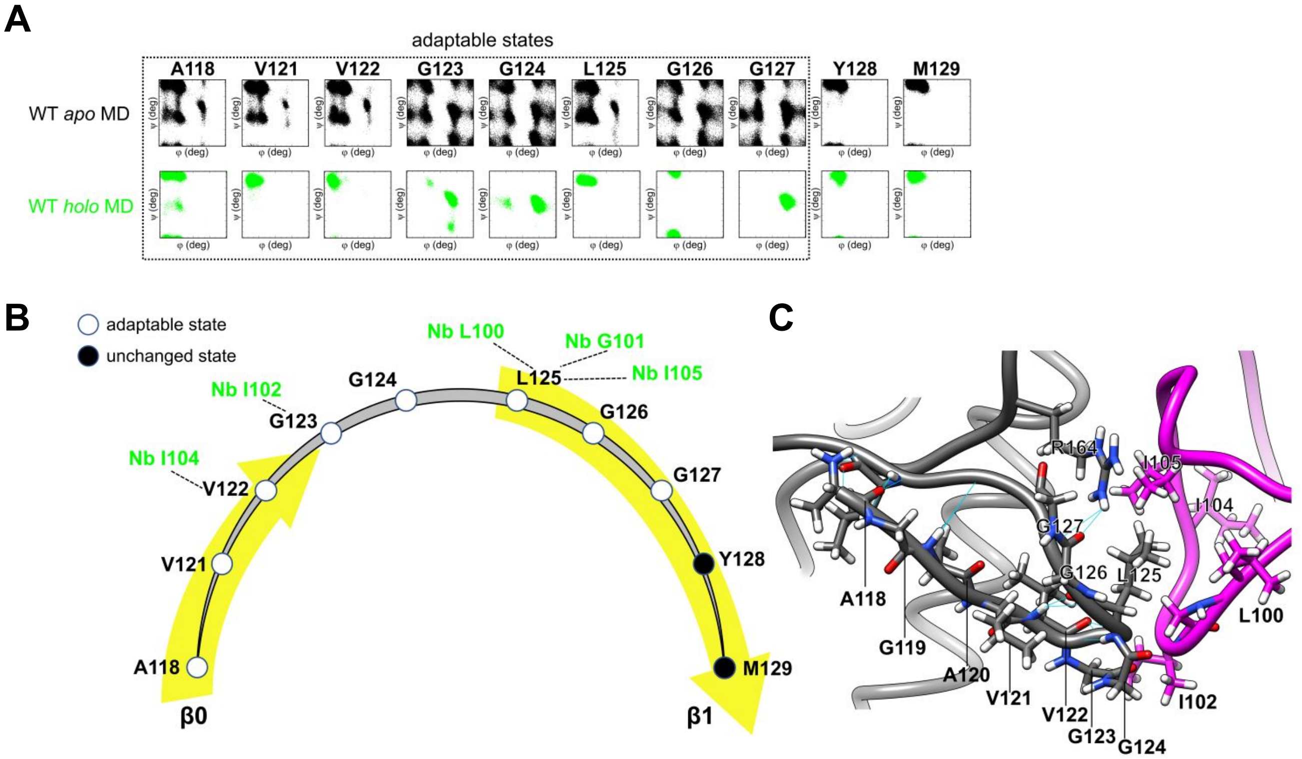

Figure S11. Mechanisms of WT HuPrP-Nb484 molecular recognition in the palindromic motif epitope region. (**A**) Ramachandran plots for selected residues within the palindromic motif. In black and green dots the *phi*/*psi* pairs from MD snapshots from *apo* MD and *holo* MD, respectively. The dotted square indicates the residues involved in Nb484 interaction and subjected to adaptable changes during the conformational selection steps. (**B**) Schematic representation of part of the palindromic motif where black and white circles represent the residues involved in unchanged or adaptable states, respectively, during the binding with key residues (indicated in green) of Nb484. (**C**) Close overview of a MD snapshot from *holo* HuPrP with highlighted the residues involved in dynamic interactions (in grey and magenta colour the HuPrP and Nb484, respectively).
